# TSP50 Attenuates DSS‐Induced Colitis by Regulating TGF‐β Signaling Mediated Maintenance of Intestinal Mucosal Barrier Integrity

**DOI:** 10.1002/advs.202305893

**Published:** 2024-01-08

**Authors:** Jiawei Li, Chunxue Niu, Huihan Ai, Xiaoli Li, Linlin Zhang, Yan Lang, Shuyue Wang, Feng Gao, Xianglin Mei, Chunlei Yu, Luguo Sun, Yanxin Huang, Lihua Zheng, Guannan Wang, Ying Sun, Xiaoguang Yang, Zhenbo Song, Yongli Bao

**Affiliations:** ^1^ NMPA Key Laboratory for Quality Control of Cell and Gene Therapy Medicine Products Northeast Normal University Changchun 130024 China; ^2^ National Engineering Laboratory for Druggable Gene and Protein Screening Northeast Normal University Changchun 130117 China; ^3^ The Key Laboratory of Molecular Epigenetics of Ministry of Education Northeast Normal University Changchun Jilin 130024 China; ^4^ Department of General Surgery Affiliated Tumor Hospital of Zhengzhou University Zhengzhou Henan 450000 China; ^5^ Department of Pathology The Second Hospital of Jilin University Changchun 130041 China

**Keywords:** IBD, intestinal barrier, TGF‐Î^2^, TSP50

## Abstract

The integrity of the intestinal mucosal barrier is crucial for protecting the intestinal epithelium against invasion by commensal bacteria and pathogens, thereby combating colitis. The investigation revealed that the absence of TSP50 compromised the integrity of the intestinal mucosal barrier in murine subjects. This disruption facilitated direct contact between intestinal bacteria and the intestinal epithelium, thereby increasing susceptibility to colitis. Mechanistic analysis indicated that TSP50 deficiency in intestinal stem cells (ISCs) triggered aberrant activation of the TGF‐β signaling pathway and impeded the differentiation of goblet cells in mice, leading to impairment of mucosal permeability. By inhibiting the TGF‐β pathway, the functionality of the intestinal mucosal barrier is successfully restored and mitigated colitis in TSP50‐deficient mice. In conclusion, TSP50 played a crucial role in maintaining the intestinal mucosal barrier function and exhibited the preventive effect against the development of colitis by regulating the TGF‐β signaling pathway.

## Graphical abstract

TSP50 deficiency, through the activation of the TGF‐β pathway, hinders goblet cell differentiation, impairs intestinal mucosal barrier integrity, and increases susceptibility to colitis. Inhibiting TGF‐β restores barrier function in TSP50‐deficient mice, alleviating colitis. This underscores the crucial role of TSP50 in maintaining mucosal integrity and preventing colitis through the regulation of TGF‐β signaling.

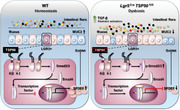

## 1 Introduction

Inflammatory bowel disease (IBD), comprising Crohn's disease (CD) and ulcerative colitis (UC), is a global issue with a rising prevalence.^[^ ^1^, ^2^ ^]^ The onset of IBD is significantly influenced by genetic factors, as evidenced by the presence of 163 shared susceptibility loci between CD and UC.^[^ ^3^, ^4^ ^]^ IBD are chronic relapsing inflammatory disorders of the gastrointestinal tract whose etiology is still unknown; the pathogenesis relies on the interplay among mainly unknown environmental factors, defects of the epithelial barrier, dysregulation of the innate and adaptive immune response at the intestinal mucosal level, and alteration of the intestinal microbiota in a genetically susceptible host.^[^ ^3^ ^]^ IBD is now regarded as a disease intricately associated with the intestinal mucosa. In contrast to the protective mucus layer in the colons of healthy individuals, which prevents bacterial penetration, the colonic mucus in patients with active UC allows bacteria to penetrate and reach the intestinal epithelium.^[^ ^1^ ^]^ Despite the importance of the intestinal mucosal barrier in intestinal diseases, the underlying molecular mechanisms governing the formation and progression of IBD remain elusive.

The primary function of the intestinal mucosal barrier is to impede the entry of detrimental substances and pathogens, thereby safeguarding the internal homeostasis of the organism.^[^ ^5^ ^]^ The secreted mucin proteins synthesized by goblet cells constitute the fundamental constituents of the mucous layer, which serves as a protective barrier for the intestinal mucosa. These mucins form a polymeric network, creating a mucus layer that separates intestinal bacteria from the host intestinal epithelium and provides protective functions. There exist two discernible classifications of mucins: gel‐forming mucins (Muc2, 5AC, 5B, 6) derived from goblet cells, and membrane‐associated mucins (Muc1, 3, 4, 13, 17) expressed by both goblet and absorptive cells.^[^ ^6^ ^]^ Studies have shown that mice lacking Muc2 (*Muc2* ^−/−^) exhibit spontaneous colitis and reduced resistance to intestinal pathogens.^[^ ^1^ ^]^ Although the significance of goblet cells in the intestinal mucosal barrier is acknowledged, the precise molecular mechanisms that govern the regulatory role of goblet cell‐derived mucus in maintaining intestinal homeostasis and preventing diseases remain elusive.

Testis‐specific protease 50 (TSP50) is a recently discovered oncogene that has been observed to exhibit elevated expression levels in a range of cancer types, including gastric cancer, colon cancer, and various others.^[^ ^7^, ^8^, ^9^, ^10^ ^]^ Furthermore, heightened expression of TSP50 has been associated with an unfavorable prognosis in gastric and colon cancers.^[^ ^11^, ^12^ ^]^ However, the precise role and underlying mechanisms of TSP50 in intestinal diseases remain poorly understood.

It has been observed that TSP50 played a vital role in regulating cell proliferation and apoptosis: increased expression promoted proliferation, while decreased expression induced apoptosis.^[^ ^7^, ^8^, ^13^, ^14^ ^]^ Additionally, TSP50 protein has been detected in both cell clusters and blastocysts during the blastocyst stage, and inhibition of TSP50 has been found to enhance cardiomyocyte differentiation in P19 cells.^[^ ^15^ ^]^ Considering the crucial requirement for self‐renewal capacity, multidirectional differentiation potential, and robust proliferative ability in intestinal tissues to maintain the integrity of the intestinal mucosal barrier, coupled with the documented high expression of TSP50 in gastric and colon cancers that correlates with an unfavorable prognosis, we hypothesize that TSP50 might be implicated in both the maintenance of the intestinal mucosal barrier and the pathogenesis of intestinal diseases.

In this study, we generated systemic and intestinal stem cell‐specific TSP50 knockout mice and employed intestinal organoid culture techniques to assess the impact of TSP50 in colitis and elucidated the physiological role of TSP50 in maintaining the intestinal mucosal barrier and preventing colitis. By specifically targeting the TSP50‐TGF‐β signaling cascade, we successfully alleviated the intestinal mucosal barrier dysfunction and colitis symptoms induced by TSP50 deficiency.

## 2 Results

### 2.1 TSP50‐Deficient Mice are Susceptible to DSS‐Induced Colitis

**Figure 1:**
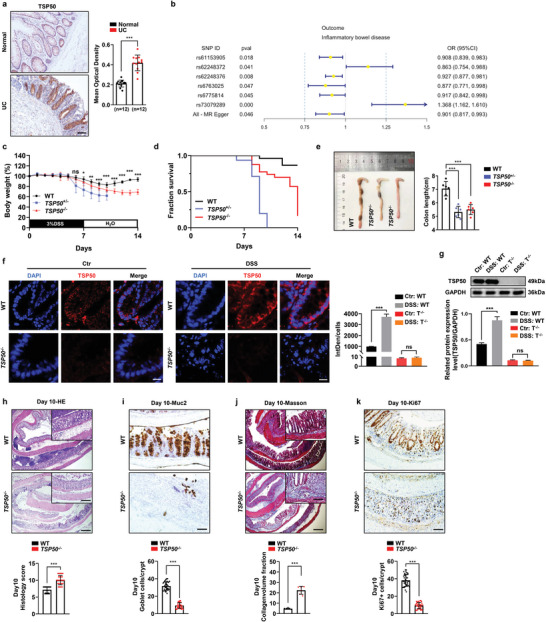
TSP50 depletion renders mice vulnerable to DSS‐induced colitis. a) Immunohistochemical assessment of TSP50 expression levels in human normal colonic tissue and colonic tissue obtained from patients with UC (Left). The mean optical density in immunohistochemical staining images of TSP50 was measured by ImageJ software (Right). Data are represented as the mean ± SD. *n* = 12, ***P < 0.001. unpaired, two‐tailed Student's *t* test). Scale bars: 50 µm. b) A two‐sample Mendelian randomization (2SMR) analysis was conducted using a database sourced from GTEx to assess the causal relationship between TSP50 and IBD. The analysis revealed a significant negative association between TSP50 expression levels and the predicted risk of developing IBD. c,d) Adult male mice were exposed to 3% dextran sodium sulfate (DSS) induction from day 0 to day 7, after which they were switched to normal drinking water from day 7 until day 14. Body weight (c) and survival rate (d) were measured and evaluated. (Data are represented as the mean ± SD, WT, *n* = 10; *TSP50* ^+/−^, *n* = 9; *TSP50* ^−/−^, *n* = 17; ns, not significant. **P < 0.05. **P < 0.01. ***P < 0.001). e) Representative images of colons obtained from WT, *TSP50* ^+/‐^ and *TSP50* ^−/−^ mice that underwent a 7‐day treatment with 3% DSS and these mice were sacrificed on day 10. (Data are represented as the mean ± SD. *n* = 6; ***P < 0.001. unpaired, two‐tailed Student's *t* test). f) Immunofluorescence assay was employed to detect the expression level of TSP50 in the colon of both normal (Ctr) mice and mice treated with 3% DSS (DSS) on day 10 (Left). Quantification of the immunofluorescence staining results by calculating the integrated density (IntDen) divided by the cell count (DAPI count) (Right). (Ctr: Normal group without DSS induction; DSS: Colitis group induced by DSS; T^−/−^: *TSP50* ^−/−^). (The Immunofluorescence results were quantitatively analyzed using ImageJ software. Data are represented as the mean ± SD. *n* = 3; ns, not significant. ***P < 0.001. unpaired, two‐tailed Student's *t* test). Scale bar: 10 µm. g) The expression level of TSP50 in the colon tissue of both WT and *TSP50* ^−/−^ mice treated with 3% DSS on day 10 was assessed using Western blotting. GAPDH was used as loading control. (Ctr: Normal group without DSS induction; DSS: Colitis group induced by DSS; T^−/−^: *TSP50* ^−/−^). (The Western blotting results were quantitatively analyzed using ImageJ software, Data are represented as the mean ± SD. *n* = 3; ns, not significant. ***P < 0.001. unpaired, two‐tailed Student's *t* test). h) Representative images of colon tissue slices stained with HE were obtained from mice treated with 3% DSS on day 10 (Top). Histological scores were evaluated in WT and *TSP50* ^−/−^ mice on day 10 post‐DSS treatment (Bottom). (Data are represented as the mean ± SD. *n* = 6; ***P < 0.001. unpaired, two‐tailed Student's *t* test). Scale bar: Entire colon section 200 µm, top right magnification 100 µm. i) The Muc2 antibody was employed in immunohistochemistry to assess the goblet cell count in the colon of mice on day 10 following treatment with 3% DSS (Top). Quantification of Muc2‐positive cells in each crypt by counting ≥6 crypts in each mouse (Bottom). (The immunohistochemistry results were quantitatively analyzed using ImageJ software. Data are represented as the mean ± SD. *n* = 6; ***P < 0.001. unpaired, two‐tailed Student's *t* test). Scale bar: 50 µm. j) Representative images of colon tissue slices stained with Masson from mice treated with 3% DSS on day 10 (Top). Masson staining results were evaluated by computing the Collagen volume fraction (CVF) (Bottom). (The Masson results were quantitatively analyzed using ImageJ software. Data are represented as the mean ± SD. *n* = 6; ***P < 0.001. unpaired, two‐tailed Student's *t* test). Scale bar: Entire colon section 200 µm, top right magnification 100 µm. k) Immunohistochemistry with the Ki67 antibody was performed to evaluate the proliferation of colon tissue in mice on day 10 after treatment with 3% DSS (Top). Quantification of Ki67‐positive cells in each crypt by counting ≥6 crypts in each mouse (Bottom). (The immunohistochemistry results were quantitatively analyzed using ImageJ software. Data are represented as the mean ± SD. *n* = 6; ***P < 0.001. unpaired, two‐tailed Student's *t* test). Scale bar: 50 µm. Data are representative of three independent experiments (a, b, and f‐k) or are pooled from two independent experiments (c–e).

First, we discovered that TSP50 was expressed in normal human intestinal tissues using the GTEx database (Figure S1a, Supporting Information), and this was further confirmed by immunohistochemical analysis in human intestines (Figure S1b, Supporting Information). The findings suggest that the regions displaying positive TSP50 expression are primarily concentrated in the intestinal crypts. To determine the association between TSP50 expression and colitis, the expression of TSP50 was assessed by immunohistochemistry in human normal colon tissue and colon tissue from patients with active UC. Our investigation unveiled that the average optical density of TSP50 in colon tissue from UC patients exhibited a significant increase when compared to that in normal intestinal epithelium (**Figure** **1**a). In addition, our result showed a negative association between the expression level of TSP50 and the risk of developing IBD (Figure 1b). In order to investigate the role of TSP50 in IBD, we generated TSP50 knockout (KO) (*TSP50* ^−/−^) mice through embryonic stem cell microinjection (Figure S1c–f, Supporting Information). And utilized a DSS‐induced colitis model in both TSP50 systemic knockout mice and wild‐type mice. Importantly, our observations demonstrated that TSP50‐deficient mice exhibited exacerbated colitis compared to gender‐matched littermate controls (Figure 1c–k). Following DSS administration, TSP50‐deficient mice experienced rapid weight loss and a significant increase in mortality rate (Figure 1c,d). Simultaneously, we observed that mice with heterozygous deletion of TSP50 exhibited more pronounced weight loss compared to mice with complete TSP50 deletion. Moreover, all the heterozygous deletion mice succumbed to DSS‐induced colitis within 14 days. Further investigation is warranted to elucidate the intricate regulatory mechanisms underlying this phenomenon. As the colon is the primary site of tissue damage induced by DSS, we assessed the expression of TSP50 and measured the colon length on day 10 following DSS treatment. We observed a significant reduction in colon length in *TSP50* ^+/−^ and *TSP50* ^−/−^ mice (Figure 1e). The expression level of TSP50 was elevated in the colon tissues of WT mice with colitis (Figure 1f,g), which was consistent with the results shown in Figure 1a. Furthermore, *TSP50* ^−/−^ mice displayed aggravated colonic epithelial injury and enhanced inflammatory infiltration, leading to higher tissue damage scores compared to WT mice after treatment with DSS (Figure 1h). Moreover, a significant decrease in goblet cells (Figure 1i; Figure S1g,h, Supporting Information), and an accumulation of collagen fibers (Figure 1j) were observed in the *TSP50* ^−/−^ mice. Notably, we observed a significant increase in the levels of cytokines (IL‐1β, TNF‐α, TGF‐β) in the intestinal tissues of *TSP50* ^−/−^ mice, in both the absence and presence of DSS treatment, when compared to WT mice (Figure S1i, Supporting Information), while no discernible damage to the colonic epithelial structures was observed (Figure S1j, Supporting Information). This indicates that there may be an inflammatory response present in the intestinal tissues of *TSP50* ^−/−^ mice even without DSS treatment. Following crypt damage caused by injuries, highly proliferative intestinal stem cells (ISCs) and progenitors undergo commitment to repair the injured tissues.^[^ ^16^ ^]^ In this regard, we investigated the proliferation of intestinal epithelial cells during the regeneration phase, characterized by the gradual restoration of crypt structure in the colon. We observed that WT mice exhibited a higher number of proliferating cells within the crypt and displayed a faster recovery compared to TSP50‐deficient mice (Figure 1k). These findings align with previous studies demonstrating the inhibitory effect of TSP50 down‐regulation on cell proliferation.^[^ ^7^, ^9^, ^14^, ^17^ ^]^ Therefore, the aggravated colitis observed in TSP50‐deficient mice following DSS‐induced colonic epithelial injury indicates that the absence of TSP50 increases the susceptibility of mice to colitis.

### 2.2 TSP50 Deficiency in ISCs Increases Susceptibility of Mice to DSS‐Induced Colitis

**Figure 2:**
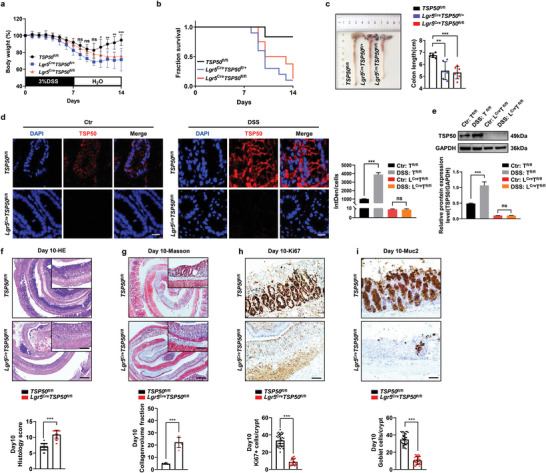
TSP50 deficiency in ISC increases mice susceptibility of colitis. a,b) Adult male mice were exposed to 3% DSS induction from day 0 to day 7, after which they were switched to normal drinking water from day 7 until day 14. Body weight (a) and survival rate (b) were measured and evaluated. (*TSP50* ^fl/fl^, *n* = 8; *Lgr5* ^Cre^ *TSP50* ^fl/+^, *n* = 7; *Lgr5* ^Cre^ *TSP50* ^fl/fl^, *n* = 10; ns, not significant. **P < 0.05. **P < 0.01. ***P < 0.001). c) Representative images of colons were obtained from *TSP50* ^fl/fl^, *Lgr5* ^Cre^ *TSP50* ^fl/+^ and *Lgr5* ^Cre^ *TSP50* ^fl/fl^ mice that underwent a 7‐day treatment with 3% DSS and were sacrificed on day 10. (Data are represented as the mean ± SD. *n* = 6; **P < 0.01. ***P < 0.001. unpaired, two‐tailed Student's *t* test). d) Immunofluorescence assay was employed to detect the expression level of TSP50 in the colon of both normal (Ctr) mice and mice treated with 3% DSS (DSS) on day 10 (Left). Quantification of the immunofluorescence staining results by calculating the integrated density (IntDen) divided by the cell count (DAPI count) (Right). (Ctr: Normal group without DSS induction; DSS: Colitis group induced by DSS; T^fl/fl^: *TSP50* ^fl/fl^; L^Cre^ T^fl/fl^: *Lgr5* ^Cre^ *TSP50* ^fl/fl^). (The immunofluorescence results were quantitatively analyzed using ImageJ software. Data are represented as the mean ± SD. *n* = 3; ns, not significant. ***P < 0.001. unpaired, two‐tailed Student's *t* test). Scale bar: 10 µm. e) The expression level of TSP50 in the colon tissue of both *TSP50* ^fl/fl^ and *Lgr5* ^Cre^ *TSP50* ^fl/fl^ mice treated with 3% DSS on day 10 was assessed using Western blotting. GAPDH was used as loading control. (Ctr: Normal group without DSS induction; DSS: Colitis group induced by DSS; T^fl/fl^: *TSP50* ^fl/fl^; L^Cre^ T^fl/fl^: *Lgr5* ^Cre^ *TSP50* ^fl/fl^). (The Western blotting results were quantitatively analyzed using ImageJ software, Data are represented as the mean ± SD. *n* = 3; ns, not significant. ***P < 0.001. unpaired, two‐tailed Student's *t* test). f) Representative images of colon tissue slices stained with HE were obtained from mice treated with 3% DSS on day 10 (Top). Histological scores were evaluated in *TSP50* ^fl/fl^ and *Lgr5* ^Cre^ *TSP50* ^fl/fl^ mice on day 10 post‐DSS treatment (Bottom). (Data are represented as the mean ± SD. *n* = 6; ***P < 0.001. unpaired, two‐tailed Student's *t* test). Scale bar: Entire colon section 200 µm, top right magnification 100 µm. g) Representative images of colon tissue slices stained with Masson from mice treated with 3% DSS on day 10 (Top). Masson staining results were evaluated by computing the Collagen volume fraction (CVF) (Bottom). (The Masson results were quantitatively analyzed using ImageJ software. Data are represented as the mean ± SD. *n* = 6; ***P < 0.001. unpaired, two‐tailed Student's *t* test). Scale bar: Entire colon section 200 µm, top right magnification 100 µm. h) Immunohistochemistry with the Ki67 antibody was performed to evaluate the proliferation of colon tissue in mice on day 10 after treatment with 3% DSS (Top). Quantification of Ki67‐positive cells in each crypt by counting ≥6 crypts in each mouse (Bottom). (The immunohistochemistry results were quantitatively analyzed using ImageJ software. Data are represented as the mean ± SD. *n* = 6; ***P < 0.001. unpaired, two‐tailed Student's *t* test). Scale bar: 50 µm. i) The Muc2 antibody was employed in immunohistochemistry to assess the goblet cell count in the colon of mice on day 10 following treatment with 3% DSS (Top). Quantification of Muc2‐positive cells in each crypt by counting ≥6 crypts in each mouse (Bottom). (The immunohistochemistry results were quantitatively analyzed using ImageJ software. Data are represented as the mean ± SD. *n* = 6; ***P < 0.001. unpaired, two‐tailed Student's *t* test). Scale bar: 50 µm. Data are representative of three independent experiments (d–i) or are pooled from two independent experiments (a, b, and c).

Substantial evidence supports the notion that intestinal stem cells, characterized by the expression of cell‐surface markers such as Lgr5, Olfm4, Ascl2, Smoc2, etc, are responsible for maintaining the renewal of homeostatic epithelial cells in the intestine. These stem cells are primarily enriched in the intestinal crypts,^[^ ^16^ ^]^ which coincides with the location of TSP50 positive expression (Figure S2a, Supporting Information). To further investigate the role of TSP50 in the intestine, a TSP50‐deficient mouse model called Lgr5‐Cre; TSP50^fl/+^ (*Lgr5* ^Cre^ *TSP50* ^fl/+^) was created by crossing Lgr5‐Cre‐ERT2 mice with *TSP50* ^fl/fl^ mice. After tamoxifen‐induced knockout of TSP50 in Lgr5+ cells within the mouse intestinal tract, survival and body weight of mice were monitored and the result suggested that the absence of TSP50 in intestinal stem cells (L*gr5* ^Cre^ *TSP50* ^fl/+^, *Lgr5* ^Cre^ *TSP50* ^fl/fl^) during DSS‐induced colitis resulted in profound weight loss and heightened mortality rates compared to the control *TSP50* ^fl/fl^ mice (**Figure** **2**a,b). It is noteworthy that *Lgr5* ^Cre^ *TSP50* ^fl/+^ mice exhibited near‐total death within 14 days following DSS administration (>80%), consistent with the results in *TSP50* ^+/−^ mice from Figure 1d. Likewise, *Lgr5* ^Cre^ *TSP50* ^fl/fl^ mice exhibited significantly diminished colonic length and pronounced intestinal congestion when compared to *TSP50* ^fl/fl^ mice following DSS treatment (Figure 2c). Consistently, in comparison to non‐DSS‐treated *TSP50* ^fl/fl^ mice, TSP50 expression was elevated after DSS treatment (Figure 2d,e). Additionally, these *Lgr5* ^Cre^ *TSP50* ^fl/fl^ mice displayed a pathological phenotype that was akin to that observed in *TSP50* ^−/−^ mice. Histological analysis by HE staining revealed that *Lgr5* ^Cre^ *TSP50* ^fl/fl^ mice manifested a nearly complete disappearance of crypt structures by day 10 (Figure 2f). Moreover, these mice displayed elevated tissue damage scores (Figure 2f), severe intestinal epithelial fibrosis (Figure 2g), inflammatory infiltration, and reduced proliferative cell population (Figure 2h). Notably, similar to *TSP50* ^−/−^ mice, *Lgr5* ^Cre^ *TSP50* ^fl/fl^ mice encountered challenges in recovery following extensive loss of goblet cells (Figure 2i; Figure S2b,c, Supporting Information). Furthermore, cytokine levels (IL‐1β, TNF‐α and TGF‐β) in the intestinal tissues of *Lgr5* ^Cre^ *TSP50* ^fl/fl^ mice were significantly elevated in both the absence and presence of DSS treatment in comparison to *TSP50* ^fl/fl^ mice (Figure S2d, Supporting Information), while no discernible damage to the colonic epithelial structures was observed (Figure S2e, Supporting Information), aligning with the findings observed in *TSP50* ^−/−^ mice. These results collectively indicate that the deficiency of TSP50 in intestinal stem cells exacerbates the severity of colitis.

### 2.3 TSP50 Deficiency Results in Reduced Number of Goblet Cells in Mice

Given the severe reduction of goblet cells observed in both *TSP50* ^−/−^ and *Lgr5* ^Cre^ *TSP50* ^fl/fl^ mice under aggravated colitis conditions, we hypothesized that TSP50 might exert an influence on colitis through its impact on goblet cells. To further investigate the influence of TSP50 deficiency on goblet cells, we conducted a quantification of the number of goblet cells within the intestinal epithelium of TSP50‐deficient mice in the absence of DSS induction.

**Figure 3:**
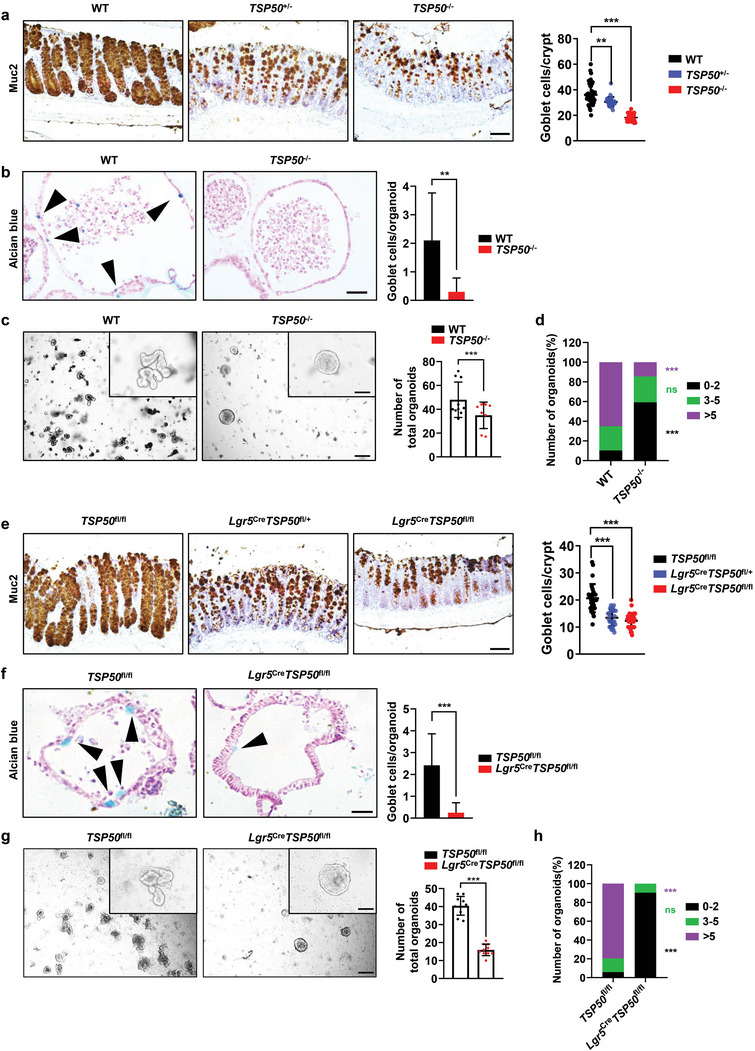
TSP50 deficiency in ISCs results in impaired differentiation of goblet cells. a) The Muc2 antibody was employed in immunohistochemistry to assess the goblet cell count in the colon of 6‐week‐old mice (Left). Quantification of Muc2‐positive cells in each crypt by counting ≥6 crypts in each mouse (Right). (The immunohistochemistry results were quantitatively analyzed using ImageJ software. Data are represented as the mean ± SD. *n* = 6; **P < 0.01. ***P < 0.001. unpaired, two‐tailed Student's *t* test). Scale bar: 50 µm. b) Goblet cells in colon organoids derived from 6‐week‐old WT and *TSP50* ^−/−^ mice were visualized using Alcian blue staining (Left). The number of Alcian blue‐positive cells was quantified (Right). (Data are represented as the mean ± SD. *n* = 6; **P < 0.01. unpaired, two‐tailed Student's *t* test). Scale bar: 30 µm. c) Representative images of colonic organoid cultures from 6‐week‐old WT and *TSP50* ^−/−^ mice on day 7, with a density of 200 crypts per well (Left). The number of colon organoids formed in 6‐week‐old WT and *TSP50* ^−/−^ mice on day 7 of culture was determined (Right). (Data are represented as the mean ± SD. *n* = 10; ***P < 0.001. unpaired, two‐tailed Student's *t* test). Scale bar: Entire images 200 µm, top right magnification 50 µm. d) The percentage of organoids with varying numbers of buds was quantified on day 7 of the organoid culture. Organoid buds number 0‐2, black; Organoid buds number 3‐5, green; Organoid buds number >5, purple. (Data are represented as the mean ± SD. *n* = 6; ns, not significant. ***P < 0.001). e) The Muc2 antibody was employed in immunohistochemistry to assess the goblet cell count in the colon of 6‐week‐old mice (Left). Quantification of Muc2‐positive cells in each crypt by counting ≥6 crypts in each mouse (Right). (The immunohistochemistry results were quantitatively analyzed using ImageJ software. Data are represented as the mean ± SD. *n* = 6; ***P < 0.001. unpaired, two‐tailed Student's *t* test). Scale bar: 50 µm. f) Goblet cells in colon organoids derived from 6‐week‐old *TSP50* ^fl/fl^ and *Lgr5* ^Cre^ *TSP50* ^fl/fl^ mice were visualized using Alcian blue staining (Left). The number of Alcian blue‐positive cells was quantified (Right). (Data are represented as the mean ± SD. *n* = 10; ***P < 0.001. unpaired, two‐tailed Student's *t* test). Scale bar: 30 µm. g) Representative images of colonic organoid cultures from 6‐week‐old *TSP50* ^fl/fl^ and *Lgr5* ^Cre^ *TSP50* ^fl/fl^ mice on day 7, with a density of 200 crypts per well (Left). The number of colon organoids formed in 6‐week‐old WT and *TSP50* ^−/−^ mice on day 7 of culture was determined (Right). (Data are represented as the mean ± SD. *n* = 10; ***P < 0.001. unpaired, two‐tailed Student's *t* test). Scale bar: Entire images 200 µm, top right magnification 50 µm. h) The percentage of organoids with varying numbers of buds was quantified on day 7 of the organoid culture. Organoid buds number 0‐2, black; Organoid buds number 3‐5, green; Organoid buds number >5, purple. (Data are represented as the mean ± SD. *n* = 10; ns, not significant. ***P < 0.001). All data are representative of at least three independent experiments.

Results from Muc2 and Alcian blue staining revealed a marked reduction in the number of goblet cells within the colon of both *TSP50* ^+/−^ and *TSP50* ^−/−^ mice (**Figure** **3**a; Figure S3a, Supporting Information). Consistently, a significant decrease in the population of goblet cells was also observed in colon organoids derived from *TSP50* ^−/−^ mice (Figure 3b; Figure S3b, Supporting Information). Notably, during the organoid culture of 6‐week‐old mice, we observed that on the 7th day of culture, the number of organoids formed by *TSP50* ^−/−^ mice was significantly reduced compared to those formed by WT mice (Figure 3c). Furthermore, the organoids derived from *TSP50* ^−/−^ mice exhibited a rounded, cystic morphology with diminished budding, resulting in a lower proportion of organoids with multiple buds (Figure 3d). Based on the result that deletion of TSP50 has a significant impact on the growth and differentiation of goblet cells within mouse intestinal organoids, which are considered as a culture system that can reflect the functionality of intestinal stem cells (ISCs) in *vivo*.^[^ ^16^ ^]^ Next, we investigated whether TSP50 affected goblet cell differentiation through its influence on ISCs. As expected, we observed a significant reduction in the number of goblet cells in both colon tissue and organoid cultures derived from *Lgr5* ^Cre^ *TSP50* ^fl/fl^ mice, which is consistent with the results observed in *TSP50* ^−/−^ mice (Figure 3e,f; Figure S3c, Supporting Information). Similarly, abnormal growth morphology and a decreased number of organoids were also observed during the organoid culture in *Lgr5* ^Cre^ *TSP50* ^fl/fl^ mice (Figure 3g,h; Figure S3d, Supporting Information). These findings collectively suggest that the deletion of TSP50 in intestinal stem cells is the primary cause for the reduction of goblet cells number in the mouse intestine.

### 2.4 TSP50 Deficiency Results in Compromised Intestinal Mucosal Barrier Function in Mice

**Figure 4:**
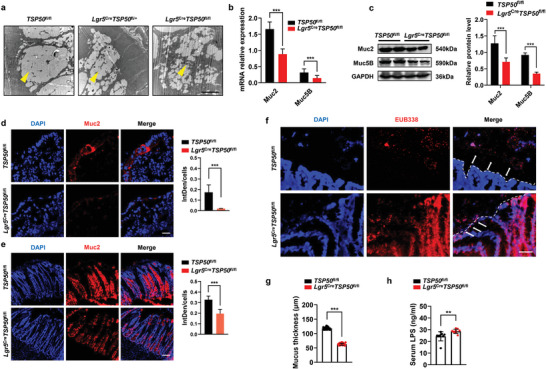
TSP50 deficiency results in compromised intestinal mucosal barrier function in mice. a) Electron microscopy was employed to investigate the ultrastructure of colonic goblet cells in mice with the *TSP50* ^fl/fl^ and *Lgr5* ^Cre^ *TSP50* ^fl/fl^ genotypes. Scale bar: 1 µm. b) The expression of Muc2 and Muc5B in the colon was detected by RT‐PCR. Values of Muc2 and Muc5B expression were normalized to those of β‐actin. (Data are presented as the mean ± SD of three independent experiments. *n* = 6; ***P < 0.001 unpaired, two‐tailed Student's *t* test). c) The expression of Muc2 and Muc5B in the colon was detected by Western blotting. GAPDH was used as loading control. (The Western blotting results were quantitatively analyzed using ImageJ software. Data are represented as the mean ± SD. *n* = 3; ***P < 0.001. unpaired, two‐tailed Student's *t* test). d,e) Immunofluorescence staining was performed to visualize the expression of the Muc2 protein in the organoid (d) and colon (e) of 6‐week‐old mice with the *TSP50* ^fl/fl^ and *Lgr5* ^Cre^ *TSP50* ^fl/fl^ genotypes (Left). Quantification of the immunofluorescence staining results by calculating the integrated density (IntDen) divided by the cell count (DAPI count) (Right). (The immunofluorescence results were quantitatively analyzed using ImageJ software. Data are represented as the mean ± SD. *n* = 3; ns, not significant. ***P < 0.001. unpaired, two‐tailed Student's *t* test). Scale bar :10 µm. f) The localization of bacteria within the inner mucus layer of the colon was assessed using fluorescence in situ hybridization (FISH) with general bacterial 16S probes (EUB 338, labeled in red). The double‐headed arrows indicate the separation between bacteria and the colonic epithelium, while the arrows indicate bacteria in close proximity to the epithelial cells. Scale bar: 50 µm. g) Measurements of total mucus thickness were conducted in the distal colon of 6‐week‐old mice with the *TSP50* ^fl/fl^ and *Lgr5* ^Cre^ *TSP50* ^fl/fl^ genotypes. (Data are represented as the mean ± SD. *n* = 6; ***P < 0.001. unpaired, two‐tailed Student's *t* test). h) Intestinal permeability was assessed by quantifying the concentration of Lipopolysaccharide (LPS) in the serum of 6‐week‐old mice with the *TSP50* ^fl/fl^ and *Lgr5* ^Cre^ *TSP50* ^fl/fl^ genotypes. (Data are represented as the mean ± SD. *n* = 6; **P < 0.01. unpaired, two‐tailed Student's *t* test). All data are representative of at least three independent experiments.

Goblet cells play a crucial role in the intestinal mucosal barrier, as they secrete mucin that forms a protective mucus layer separating the intestinal flora from the epithelium.^[^ ^1^ ^]^ To investigate the impact of TSP50 deficiency on goblet cell function, we examined the ultrastructure of goblet cells using transmission electron microscopy. We observed that the mucus‐secreting vesicles within goblet cells in the small intestine and colon of *Lgr5* ^Cre^ *TSP50* ^fl//+^ and *Lgr5* ^Cre^ *TSP50* ^fl/fl^ mice appeared atrophied, indicating potential impairment of the secretory function of goblet cells (**Figure** **4**a). Subsequently, we assessed the expression levels of mucin produced by goblet cells. Our results revealed a significant reduction in mucin expression in both the intestine and organoid cultures of *Lgr5* ^Cre^ *TSP50* ^fl/fl^ mice (Figure 4b–e; Figure S4a, Supporting Information). Notably, Muc2‐deficient mice (*Muc2* ^−/−^) have been reported to develop spontaneous colitis due to altered colonic mucus permeability.^[^ ^1^ ^]^ To evaluate the intestinal mucosal permeability in *Lgr5* ^Cre^ *TSP50* ^fl/fl^ mice in the absence of induced DSS, we performed in situ hybridization. The results indicated that the colonic mucus layer in *Lgr5* ^Cre^ *TSP50* ^fl/fl^ mice allowed bacteria to penetrate and come into contact with the epithelium (Figure 4f), which may contribute to the increased levels of inflammatory factors observed in the colon tissues of TSP50‐deficient mice without DSS induction. Additionally, we measured the total mucus thickness in the distal colon of adult *TSP50* ^fl/fl^ and *Lgr5* ^Cre^ *TSP50* ^fl/fl^ mice, and found that there was a significant reduction in the thickness of mucus layer in *Lgr5* ^Cre^ *TSP50* ^fl/fl^ mice (Figure 4g). Furthermore, the results demonstrated a significant increase in serum LPS levels in *Lgr5* ^Cre^ *TSP50* ^fl/fl^ mice (Figure 4h), indicating that TSP50 deficiency led to increased intestinal mucosal permeability. In summary, our study suggests that the absence of TSP50 results in a reduction of goblet cells, disruption of the mucus layer, allowing direct contact between the gut microbiota and intestinal epithelium, leading to elevated expression levels of inflammatory factors.

### 2.5 TSP50 Deficiency in the Intestine Impairs the Differentiation of Goblet Cells Through the Upregulation of TGF‐β Signaling

Previous studies have provided evidence linking TSP50 to the TGFβ superfamily,^[^ ^9^ ^]^ and the role of TGF‐β in regulating goblet cell differentiation and influencing the growth of intestinal organoids have been demonstrated.^[^ ^18^, ^19^ ^]^ Therefore, we have reason to speculate that TSP50 may be involved in the regulation of intestinal stem cell differentiation through the TGF‐β signaling pathway.

**Figure 5:**
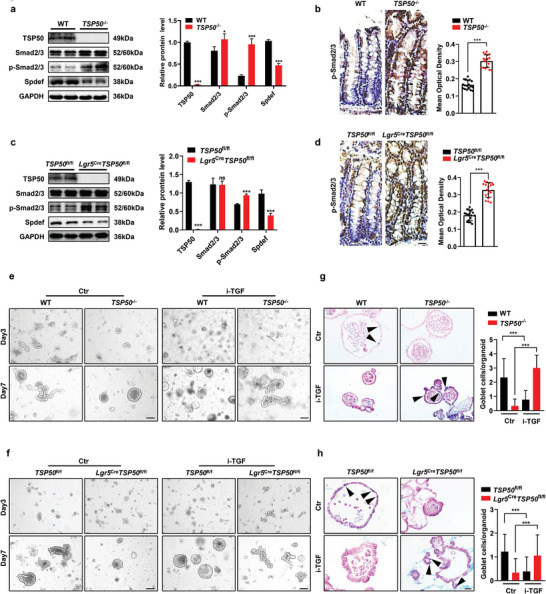
TSP50 deficiency in the intestine impairs the differentiation of goblet cells through the upregulation of TGF‐β signaling. a) The expression levels of TGF‐β signaling‐related proteins, including Smad2/3, p‐Smad2/3, and Spdef in the colon tissues of 6‐week‐old WT and *TSP50* ^−/−^ mice were assessed by Western blotting. GAPDH was used as loading control. (The Western blotting results were quantitatively analyzed using ImageJ software, Data are represented as the mean ± SD. *n* = 3; *P < 0.05. ***P < 0.001. unpaired, two‐tailed Student's *t* test). b) The expression of p‐Smad2/3 in the colon tissues of 6‐week‐old WT and *TSP50* ^−/−^ mice was evaluated by immunohistochemistry (Left). The mean optical density in immunohistochemical staining images of p‐Smad2/3 was measured by ImageJ software (Right). (The immunohistochemical results were quantitatively analyzed using ImageJ software. Data are represented as the mean ± SD. *n* = 6, ***P < 0.001. unpaired, two‐tailed Student's *t* test). Scale bar :20 µm. c) The expression levels of TGF‐β signaling‐related proteins, including Smad2/3 and p‐Smad2/3 in the colon tissues of 6‐week‐old *TSP50* ^fl/fl^ and *Lgr5* ^Cre^ *TSP50* ^fl/fl^ mice were assessed by Western blotting. GAPDH was used as loading control. (The Western blotting results were quantitatively analyzed using ImageJ software, Data are represented as the mean ± SD. *n* = 3; ns, not significant. ***P < 0.001. unpaired, two‐tailed Student's *t* test). d) The expression of p‐Smad2/3 in the colon tissues of 6‐week‐old *TSP50* ^fl/fl^ and *Lgr5* ^Cre^ *TSP50* ^fl/fl^ mice was evaluated by immunohistochemistry (Left). The mean optical density in immunohistochemical staining images of p‐Smad2/3 was measured by ImageJ software (Right). (The immunohistochemical results were quantitatively analyzed using ImageJ software, Data are represented as the mean ± SD. *n* = 6, ***P < 0.001. unpaired, two‐tailed Student's *t* test). Scale bar :20 µm. e) Representative morphological images of colon organoid cultures from 6‐week‐old WT and *TSP50* ^−/−^ mice on the 3rd and7th day of culture. The cultures were treated with the control agent (Ctr) or the TGF‐β signaling pathway inhibitor GW788388 (i‐TGF) at a concentration of 5 µm. Scale bar :100 µm. f) Representative morphological images of colon organoid cultures from 6‐week‐old *TSP50* ^fl/fl^ and *Lgr5* ^Cre^ *TSP50* ^fl/fl^ mice on the 3rd and 7th day of culture. The cultures were treated with the control agent (Ctr) or the TGF‐β signaling pathway inhibitor GW788388 (i‐TGF) at a concentration of 5 µm. Scale bar :100 µm. g) After a 7‐day treatment with a TGF‐β inhibitor, the defective differentiation of goblet cells in colon organoids derived from 6‐week‐old *TSP50* ^−/−^ mice was restored. The organoid sections were stained with Alcian Blue to label the goblet cells (Left). The number of positively stained cells was quantified (Right). (Data are represented as the mean ± SD. *n* = 6; ***P < 0.001. unpaired, two‐tailed Student's *t* test). Scale bar: 50 µm. h) After a 7‐day treatment with a TGF‐β inhibitor, the defective differentiation of goblet cells in colon organoids derived from 6‐week‐old *Lgr5* ^Cre^ *TSP50* ^fl/fl^ mice was restored. The organoid sections were stained with Alcian Blue to label the goblet cells (Left). (Data are represented as the mean ± SD. *n* = 6; ***P < 0.001. unpaired, two‐tailed Student's *t* test). The number of positively stained cells was quantified (Right). Scale bar: 50 µm. All data are representative of at least three independent experiments.

We first examined the levels of key proteins involved in the TGF‐β signaling pathway in the intestinal tissues of 6‐week‐old mice and observed that TSP50‐deficient mice exhibited significantly higher levels of phosphorylated Smad2/3 and increased nuclear translocation of Smad3 and phosphorylated Smad2/3 compared to control mice (**Figure** **5**a–d; Figure S5a,b, Supporting Information). Additionally, we observed a significant decrease in the expression levels of Spdef (a downstream target gene of the TGF‐β signaling pathway) (Figure 5a,c). These findings suggest that the loss of TSP50 may impact goblet cell differentiation and maintenance of the intestinal mucosal barrier through the activation of the TGF‐β signaling pathway. To confirm that aberrant activation of the TGF‐β signaling pathway was responsible for TSP50 loss induced impairment of intestinal mucosal barrier, we treated the organoid cultures with inhibitors of the TGF‐β signaling pathway. Surprisingly, the addition of a TGF‐β signaling pathway inhibitor (i‐TGF: GW788388, TGFβRI‐IN‐1) restored the impaired growth morphology of TSP50‐deficient organoids (Figure 5e,f; Figure S5c–l, Supporting Information). Additionally, the presence of the TGF‐β signaling pathway inhibitor (GW788388, TGFβRI‐IN‐1) also resulted in altered growth morphology of intestinal organoids in control WT and *TSP50* ^fl/fl^ mice (Figure 5e,f; Figure S5c–l, Supporting Information), indicating that the TGF‐β signaling pathway plays a key role in the growth, renewal and differentiation of intestinal organoids (2.5 µm, 5 µm, and 10 µm of inhibitors were used for the assays; only representative images are shown at the concentration 5 µm; other data are not shown). Notably, the number of goblet cells in *TSP50* ^−/−^ mice intestinal organoids increased significantly after 7 days of GW788388 addition (Figure 5g). However, intriguingly, a significant reduction of goblet cells number was observed in the intestinal organoids of 6W‐WT mice after the same treatment (Figure 5g) and comparable results were observed in the cultivation of intestinal organoids derived from 6‐week‐old *Lgr5* ^Cre^ *TSP50* ^fl/fl^ mice (Figure 5h). These findings indicate that both excessive activation and suppression of the TGF‐β signaling pathway can result in aberrant differentiation of goblet cells in adult mouse intestinal organoids. Overall, our findings highlight the importance of TSP50 in modulating the TGF‐β signaling pathway and emphasize its role in maintaining the balance of this pathway for correct goblet cell differentiation in the intestine.

### 2.6 TSP50 Interacts with TGFβRII to Modulate TGF‐β Downstream Signaling

TGF‐β superfamily ligands bind to type II receptors, leading to the phosphorylation and activation of type I receptors. The activated type I receptors then phosphorylate R‐Smads, which form complexes with co‐Smad (Smad4) and translocate into the nucleus. In the nucleus, these complexes regulate the expression of downstream genes.^[^ ^20^, ^21^, ^22^ ^]^

**Figure 6:**
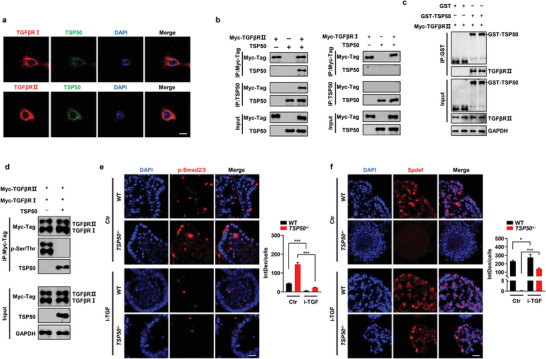
TSP50 directly binds to TGFβRII and inhibits its phosphorylation, thereby regulating TGF‐β signaling. a) TSP50 colocalized with TGFβRII and TGFβRI in NCM 460 cells. Scale bars: 10 µm. b) Coexpression of pcMV‐myc‐TGFβRII or pcMV‐myc‐TGFβRI with pcDNA3.1‐TSP50 was performed in HEK 293T cells. Cell extracts were subjected to immunoprecipitation using an anti‐myc tag antibody for detection of TGFβRII and TGFβRI, followed by Western blotting analysis using an anti‐TSP50 antibody. Additionally, reciprocal co‐immunoprecipitation (Co‐IP) between TSP50 and TGFβRII or TGFβRI was carried out in HEK 293T cells. c) Proteins purified from B21 bacteria, including GST‐TSP50 and Myc‐TGFβRII, were subjected to a GST pull‐down assay. The interaction between Myc‐TGFβRII and GST‐TSP50 was assessed, followed by Western blotting analysis using anti‐TGFβRII and anti‐GST antibodies. d) Coexpression of pcMV‐myc‐TGFβRII or pcMV‐myc‐TGFβRI was performed with either pcDNA3.1 vector or pcDNA3.1‐TSP50 in HEK 293T cells. Cell extracts were subjected to immunoprecipitation using an anti‐myc tag antibody, which was used for the detection of TGFβRII and TGFβRI. Subsequently, Western blotting analysis was carried out using anti‐TSP50 antibody and anti p‐Ser/Thr antibodies. e,f) After a 7‐day treatment with a TGF‐β inhibitor, immunofluorescence analysis was performed to examine the expression of p‐Smad2/3 (e) and Spdef (f) in colon organoids derived from 6‐week‐old WT and *TSP50* ^−/−^ mice (Left). Quantification of the immunofluorescence staining results by calculating the integrated density (IntDen) divided by the cell count (DAPI count) (Right). (The immunofluorescence results were quantitatively analyzed using ImageJ software. Data are represented as the mean ± SD. *n* = 3; ns, not significant. ***P < 0.001. unpaired, two‐tailed Student's *t* test). Scale bar :20 µm. All data are representative of at least three independent experiments.

To further investigate the molecular mechanisms underlying the aberrant TGF‐β signaling caused by TSP50 deficiency, we performed immunofluorescence analysis in NCM 460 cells. The results revealed that TSP50 co‐localized with TGFβRI/TGFβRII on the cell membrane, indicating a potential interaction between TSP50 and the TGF‐β receptor (**Figure** **6**a). This observation suggests that TSP50 may play a role in modulating the activity of the TGF‐β signaling pathway by directly interacting with the TGF‐β receptor complex. To provide additional evidence of the interaction between TSP50 and the TGF‐β receptor, we performed co‐immunoprecipitation assays in HEK 293T cells. The results demonstrated an interaction between TSP50 and TGFβRII, while no interaction was observed between TSP50 and TGFβRI (Figure 6b). Furthermore, we conducted a GST pull‐down assay to directly assess the binding of TSP50 to TGFβRII and the results confirmed that TSP50 bound directly to TGFβRII, providing additional support for the interaction between these two proteins (Figure 6c). Taken together, these findings indicate that TSP50 specifically interacts with TGFβRII, potentially contributing to the modulation of TGF‐β signaling pathway activation. Furthermore, our results suggested that TSP50 strongly inhibited the phosphorylation of both TGFβRI and TGFβRII (Figure 6d). As phosphorylation of TGFβRI directly influences the nuclear translocation of Smad2/3, we further demonstrated that the levels of phosphorylated Smad2/3 were significantly elevated in intestinal organoids derived from 6‐week‐old *TSP50* ^−/−^ mice through immunofluorescence staining (Figure 6e). To investigate the functional implications of TGF‐β signaling pathway overactivation, we added GW788388, an inhibitor of TGF‐β signaling (i‐TGF), to the intestinal organoid culture. And, we observed that the expression levels of Spdef in organoids derived from i‐TGF‐treated *TSP50* ^−/−^ mice showed partial restoration when compared to organoids formed by *TSP50* ^−/−^ mice in the Ctr group (Figure 6f). These findings suggest that the interaction between TSP50 and TGFβRII is essential for maintaining the balance of TGF‐β signaling in the intestine.

### 2.7 TGF‐β Inhibitors Alleviate DSS‐Induced Colonic Inflammation by Restoring Intestinal Mucosal Barrier Function in TSP50‐Deficient Mice

**Figure 7:**
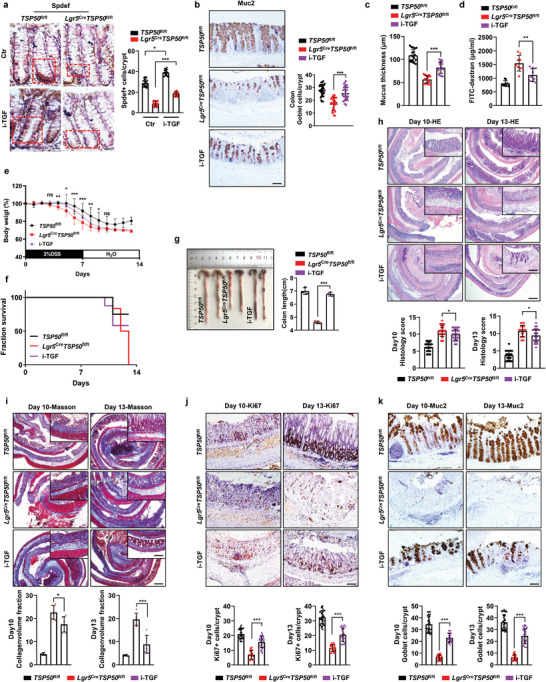
Restoring intestinal barrier function and alleviating colitis through inhibition of TGF‐β signaling in TSP50‐deficient mice. a) Representative immunohistochemical images of Spdef staining in colon tissues of *TSP50* ^fl/fl^ and *Lgr5* ^Cre^ *TSP50* ^fl/fl^ mice, following 5‐day oral administration of a TGF‐β inhibitor (i‐TGF: 10 mg kg^−1^) or control agent. Positive staining regions are indicated by red dashed lines (Left). Quantification of Spdef‐positive cells in each crypt by counting ≥6 crypts in each mouse (Right). (The immunohistochemical results were quantitatively analyzed using ImageJ software. Data are represented as the mean ± SD. *n* = 6; *P < 0.05. ***P < 0.001. unpaired, two‐tailed Student's *t* test). Scale bars: 20 µm. b) Representative immunohistochemical staining of Muc2 for goblet cells in colon tissues of *Lgr5* ^Cre^ *TSP50* ^fl/fl^ mice, following 5‐day oral administration of a TGF‐β inhibitor (i‐TGF: 10 mg k^−1^g) or control agent (Left). The number of staining‐positive cells was quantified (Right); 3–5 crypts per mouse were examined, *n* = 6 mice per group. (Data are represented as the mean ± SD. *n* = 6; ***P < 0.001. unpaired, two‐tailed Student's *t* test). Scale bars: 50 µm. c) Total mucus thickness in the distal colon of *TSP50* ^fl/fl^ and *Lgr5* ^Cre^ *TSP50* ^fl/fl^ mice (with or without inhibitor) was assessed after 5‐day oral administration of a TGF‐β (i‐TGF: 10 mg kg^−1^) inhibitor or control agent. (Data are represented as the mean ± SD. *n* = 6; ***P < 0.001. unpaired, two‐tailed Student's *t* test). d) Intestinal permeability was assessed by measuring the concentration of FITC‐dextran in the serum of *TSP50* ^fl/fl^ and *Lgr5* ^Cre^ *TSP50* ^fl/fl^ mice after treatment with a TGF‐β inhibitor (i‐TGF: 10 mg kg^−1^) or control agent. (Data are represented as the mean ± SD. n = 6; ***P < 0.001. unpaired, two‐tailed Student's *t* test). e, f) The percentage of body weight loss (e) and the survival rate (f) of *TSP50* ^fl/fl^ and *Lgr5* ^Cre^ *TSP50* ^fl/fl^ mice (with or without TGF‐β inhibitor) was examined after 5‐day oral administration of a TGF‐β inhibitor (i‐TGF: 10 mg kg^−1^) or control agent, followed by induction of colitis using 3% DSS. (Data are represented as the mean ± SD, *TSP50* ^fl/fl^, *n* = 6; *Lgr5* ^Cre^ *TSP50* ^fl/fl^, n = 6; i‐TGF, n = 7; *Lgr5* ^Cre^ *TSP50* ^fl/fl^ as i‐TGF control. ns, not significant. *P < 0.05. **P < 0.01. ***P < 0.001). g) After a 5‐day oral administration of a TGF‐β inhibitor (i‐TGF: 10 mg k^−1^g) or control agent, colitis was induced using 3% DSS. On the 13th day of colitis induction, colonic length was measured. (Data are represented as the mean ± SD. *n* = 3; ***P < 0.001. unpaired, two‐tailed Student's *t* test). h–j) After a 5‐day oral administration of a TGF‐β inhibitor (i‐TGF: 10 mg kg^−1^) or control agent, colitis was induced using 3% DSS. On the 10th and 13th days of colitis induction, representative images of colon tissue sections were obtained following staining with HE (h), Masson (i), and Ki67 (j) (Top). Histological scores were evaluated in *TSP50* ^fl/fl^ and *Lgr5* ^Cre^ *TSP50* ^fl/fl^ mice on day 10, 13 post‐DSS treatment (h, Bottom). Masson staining results were evaluated by computing the Collagen volume fraction (CVF) (i, Bottom). Quantification of Ki67‐positive cells in each crypt by counting ≥6 crypts in each mouse (j, Bottom). (Data are represented as the mean ± SD. *n* = 3; *P < 0.05. ***P < 0.001. unpaired, two‐tailed Student's *t* test). Scale bar: Entire colon section 200 µm, top right magnification 100 µm (h,i). 50 µm (j). k) After 5 days of oral administration of a TGF‐β inhibitor (i‐TGF: 10 mg kg^−1^) or control agent, colitis was induced using 3% DSS. On the 10th and 13th days of colitis induction, colonic tissue sections were immunohistochemically stained with a Muc2 antibody to quantify goblet cell numbers (Top). Quantification of Muc2‐positive cells in each crypt by counting ≥6 crypts in each mouse. (The immunohistochemistry results were quantitatively analyzed using ImageJ software. Data are represented as the mean ± SD. *n* = 6; ***P < 0.001. unpaired, two‐tailed Student's *t* test). Scale bar: 50 µm. Data are representative of three independent experiments (a‐d and h‐k) or are pooled from two independent experiments (e–g).

To investigate whether inhibition of TGF‐β signaling could rescue the impaired goblet cell differentiation in TSP50‐deficient mice, we examined the regulatory effect of TGF‐β on the expression of *Spdef*, a transcription factor involved in goblet cell differentiation. Previous studies have demonstrated that TGF‐β can directly inhibit the transcription of *Spdef*, and inhibition of *Spdef* using shRNAs leads to reduced expression of genes such as *AGR2*, *Muc2*, *RETLNB*, and *SPINK4* in goblet cells.^[^ ^18^, ^23^ ^]^ After treating *Lgr5* ^Cre^ *TSP50* ^fl/fl^ mice with a TGF‐β inhibitor (i‐TGF), the expression level of Spdef was restored (**Figure** **7**a; Figure S6a, Supporting Information). Furthermore, a significant increase in the number of goblet cells in the intestine, restoration of Muc2 expression levels (Figure 7b; Figure S6b, Supporting Information) and partial restoration of intestinal mucus layer thickness were also observed (Figure 7c), with no significant changes in body weight (Figure S6c, Supporting Information). By performing the FITC‐D intestinal mucosal permeability assay, we observed that the administration of the TGF‐β inhibitor resulted in a partial restoration of intestinal mucosal permeability in *Lgr5* ^Cre^ *TSP50* ^fl/fl^ (Figure 7d). Further, the inhibitor‐treated *Lgr5* ^Cre^ *TSP50* ^fl/fl^ mice (i‐TGF) exhibited a notable reduction in the percentage of weight loss during Day 5–10, in contrast to the untreated *Lgr5* ^Cre^ *TSP50* ^fl/fl^ mice (Figure 7e). Moreover, there was a significant increase in survival rates during the colitis period, accompanied by a reduction in colon shortening (Figure 7f,g). Compared to *TSP50* ^fl/fl^ mice, the *Lgr5* ^Cre^ *TSP50* ^fl/fl^ mice treated with the TGF‐β inhibitor exhibited a certain degree of alleviation in intestinal epithelial injury and collagen fiber accumulation as well as increased proliferating cell numbers (Figure 7h–j). Most importantly, the loss of goblet cells was mitigated, and the levels of associated cytokine expression were restored (Figure 7k; Figure S6d,e, Supporting Information). Moreover, similar experimental results were obtained in *TSP50* ^−/−^ mice (Figure S6g–n, Supporting Information). The results suggest that inhibiting the abnormal activation of TGF‐β signaling caused by TSP50 deficiency contributes to alleviating colitis in DSS‐induced TSP50‐deficient mice.

## 3 Discussion

Although aberrant TSP50 gene expression has been reported in several types of cancer, the role of TSP50 in IBD, specifically UC, has not been extensively studied. In the present study, we aimed to investigate the potential involvement of TSP50 in IBD. We analyzed clinical samples from patients with active UC and found that TSP50 was highly expressed in the colonic tissue of these patients. Based upon this observation, we investigated the role of TSP50 in colitis using mice with systemic and intestinal stem cell‐specific knockout of TSP50 and found that these mice showed increased susceptibility to DSS‐induced colitis.

TSP50 exhibited substantial upregulation in diverse malignancies, including gastric, colorectal, and mammary neoplasms and it played pivotal roles in a wide array of biological processes, comprising tumor cell proliferation, apoptotic events, and metabolic activities.^[^ ^7^, ^8^, ^9^, ^10^, ^24^ ^]^ Fascinatingly, our investigation revealed that heterozygous mice exhibited a greater propensity for developing exacerbated colitis compared to their pure heterozygous counterparts, as observed in both systemic and intestinal stem cell‐specific TSP50 knockout mouse models. This intricate cascade of responses, potentially instigated by the intermediate expression of proteins in heterozygous mice, encompasses a multitude of factors, including but not limited to genetic compensation^[^ ^25^ ^][^ ^26^ ^][^.^27^ ^]^ Further studies are needed to elucidate the detailed mechanisms underlying the heterozygous deletion of TSP50.

In this study, we made a notable discovery that TSP50‐deficient mice exhibited a substantial reduction in goblet cell population within a DSS‐induced colitis model. In order to ascertain whether the decline in goblet cells was the cause or consequence of colitis exacerbation, we examined indicators associated with goblet cells and intestinal mucosal barrier functionality in mice without DSS treatment. Remarkably, we observed not only a decrease in goblet cell count in the intestine of TSP50‐deficient mice but also the presence of mucus vesicle atrophy within goblet cells, alongside impaired intestinal mucosal barrier function (Figures 1, 2, 3, 4). Such alterations in the intestinal mucus layer could potentially lead to dysbiosis and compromise the integrity of the intestinal barrier, thereby predisposing the host to a range of complications.^[^ ^28^ ^]^ To determine the direct impact of TSP50 deficiency on the intestine, we ensured that all experimental mice were aged 6 weeks or older. Additionally, the induction of TSP50 knockout specifically in intestinal stem cells was carried out at the age of 6 weeks. This approach helped us ascertain that the reduction in goblet cell numbers was a direct consequence of TSP50 depletion in the intestine, rather than an indirect result of TSP50 knockout affecting the growth and development of the mice.

In our organoid experiments, we observed a notable decrease in the population of goblet cells and a significant reduction in Muc2 expression in *TSP50* ^−/−^ and *Lgr5* ^Cre^ *TSP50* ^fl/fl^ mice (Figure 4). This revelation unveils the pivotal role of TSP50 in the differentiation of goblet cells. Remarkably, TSP50‐deficient mice exhibited similar goblet cell defects to those observed in *Muc2*,^[^ ^1^, ^29^ ^]^ *FOXO1*,^[^ ^30^ ^]^ *KLF4* ^[^ ^31^ ^]^ and *SPDEF* ^[^ ^18^, ^32^ ^]^ deficient mice, making them more susceptible to DSS‐induced colitis. Interestingly, previous studies have demonstrated that the loss of TGF‐β signaling results in the expansion of goblet cells and the proliferation of conjunctiva epithelial cells.^[^ ^18^ ^]^ Consistent with these findings, our study revealed that TSP50 deficiency led to aberrant activation of TGF‐β signaling, which inhibited goblet cell differentiation and maturation. This suggests that the appropriate activation of TGF‐β signaling is crucial for maintaining the integrity of the intestinal mucosal barrier. Correspondingly, we have observed a decrease in the number of goblet cells in the intestinal tract of TSP50‐deficient mice, indicating an abnormality in goblet cell differentiation. Our previous investigations have demonstrated that TSP50 promotes cell proliferation by suppressing the activity of activins, members of the TGF‐β superfamily.^[^ ^9^ ^]^ Consistently, the absence of TSP50 leads to anomalous activation of the TGF‐β signaling pathway. Worth noting, the inhibition of the TGF‐β signaling pathway restores the growth morphology defects observed in the adult *TSP50* ^−/−^ and *Lgr5* ^Cre^ *TSP50* ^fl/fl^ mice's organoids, and rescues the impaired goblet cell differentiation within the organoids (Figure 5).

The TGF‐β superfamily plays a crucial role in regulating various physiological processes, ranging from development to disease onset.^[^ ^33^ ^]^ The activity of TGF‐β signaling increases as intestinal stem cells differentiate and migrate toward the intestinal lumen, reaching its peak in the middle region of the crypts before gradually declining.^[^ ^19^ ^]^ In colon cancer, more than 55% of cases exhibit mutations in the TGF‐β receptor type II (TGF‐βRII) .^[^ ^34^ ^]^ In our study, we have discovered a direct interaction between TSP50 and TGFβRII, whereby TSP50 inhibits the phosphorylation of TGFβRI and TGFβRII, consequently affecting the phosphorylation and nuclear translocation of Smad2/3. This ultimately regulates the expression of target genes (Figure 6), impacting goblet cell differentiation and mucin secretion, and ultimately influencing the maintenance of intestinal mucosal barrier integrity. Interestingly, in the inflamed intestine of patients with IBD, TGF‐β1 expression is upregulated but TGF‐β1–mediated immunosuppression is markedly impaired because of high Smad7, an intracellular inhibitor of TGF‐β1–associated signaling.^[^ ^35^ ^]^ Consistently, knockdown of Smad7 with a specific antisense oligonucleotide restores TGF‐β1 activity, thus leading to decreased production of inflammatory cytokines in both colitic mice and IBD patients and attenuates clinical activity in Crohn's disease patients.^[^ ^35^ ^]^ Therefore, further research is needed to fully understand the specific mechanisms through which TSP50 influences the pathogenesis of IBD and to explore potential therapeutic strategies targeting TSP50 or the TGF‐β signaling pathway in IBD treatment.

In conclusion, our study provides evidence that TSP50 influences goblet cell differentiation and mucus secretion through TGF‐β signaling, ultimately impacting the integrity of the epithelial barrier. In contrast to prior investigations, our study offers a comprehensive analysis of the in *vivo* function and mechanism of TSP50, highlighting that TSP50 deficiency‐mediated dysfunction of the intestinal mucosal barrier and worsened colitis can be mitigated through the inhibition of TGF‐β signaling. Our findings provide novel insights into the understanding of inflammatory bowel disease and suggest that targeting TGF‐β signaling activity in individuals with TSP50 deficiency may contribute to enhancing mucous protection and preventing/alleviating colitis.

## 4 Experimental Section

### Antibodies and Reagents

Primary antibodies used in this study were rabbit anti‐TSP50 (Abcam, ab181993), rat anti‐TSP50 (Novus, 260 514), rabbit anti‐β‐catenin (Abcam, ab223075), rabbit anti‐TGFβRI (Abcam, ab235578), rabbit anti‐TGFβRII (Abcam, ab259360) rabbit anti‐smad2/3 (Cell Signaling Technology, 8685), rabbit anti‐p‐Smad2/3 (Cell Signaling Technology, 8828), rabbit anti‐Spdef (LSBio, 211 521), rabbit anti‐Ki67 (ZSGB‐BIO, ZA‐0502). Secondary antibodies kits for immunohistochemical (IHC) and DAB staining kit were purchased from ZSGB‐BIO (Beijing, China). FITC‐conjugated goat anti‐rat IgG antibody, Cyanine3 (Cy3)‐conjugated goat anti‐rabbit IgG antibody for immunofluorescence (IF),4,6‐diamidino‐2‐phenylindole (DAPI) and X‐gal staining kit were purchased from Beyotime Institute of Biotechnology.

Hematoxylin and eosin (HE), Masson, Alcian Blue, Nuclear fast red staining kits and Carnoy's Fluid were purchased from Solarbio (Solarbio, Beijing, China). For organoid culture, Dulbecco's Modified Eagle Medium: F‐12 (DMEM/F12) medium, Mouse IntestiCult Organoid Growth Medium, Gentle Cell Dissociation Reagent, Dulbecco's Phosphate Buffered Saline (DPBS), and frozen stock solution were purchased from Stem cell Technologies(Vancouver, BC, Canada). Matrigel was from Corning (corning, NY, USA). 70‐µm cell strainers were from BD Bioscience (BD Bioscience, NA, USA).

### Sensitivity Analysis

Two‐sample mendelian randomization analysis was performed using the R package TwoSampleMR (v.0.5.6) and the in‐house R scripts used to perform 2SMR analysis and generate figures were available on GitHub (https://github.com/evyforjazz/2SMR). MR‐Egger regression method was performed to test the horizontal pleiotropy and heterogeneity. Exposure data of the TSP50 features containing over 3 SNPs could be used for MR‐Egger regression analysis (GTEx Database). After data harmonization and causal effect evaluation, a sensitivity analysis was conducted to predict the causal relationship between TSP50 and inflammatory bowel disease. The detailed method description is as mentioned earlier.^[^ ^36^ ^]^

### Animals

All experimental procedures were reviewed and approved by the Animal Advisory Committee at Northeast Normal University, China. The standard of the laboratory condition was specific pathogen‐free (SPF). All of the mice were maintained under a 12‐hour light/12‐hour dark cycle (lights on at 6 a.m. and lights off at 6 p.m.). The mice were mated, bred, and genotyped in the Animal Experimental Center of National Engineering Laboratory for Druggable Gene and Protein Screening. All of the mice used in this study were maintained on a C57BL/6N background.

TSP50 heterozygous Knockout (KO) (*TSP50* ^+/−^) mice were generated by the GemPharmatech Co., Ltd (Nanjing, China). A pair of loxP sites were inserted, flanking TSP50 exon 2, and a LacZ‐neomycin cassette that flanked FRT was inserted in intron 1, which terminated TSP50 transcription. Homozygous *TSP50* ^−/−^ mice were generated by crossing *TSP50* ^+/−^ mice. *TSP50* ^−/−^ mice and WT littermates were used for further experiments.

The knockout‐first mice were then bred with Flp recombinase transgenic mice (completed by GemPharmatech Co., Ltd) to remove the LacZ‐neomycin cassette, yielding *TSP50* ^fl/+^ heterozygous TSP50 conditional knockout mice that contained the TSP50 allele with exon 2 flanked by LoxP sites. Homozygous *TSP50* ^fl/fl^ mice were generated by crossing *TSP50* ^fl/+^ mice. *TSP50* ^fl/fl^ mice were then bred with the B6/JNju‐Lgr5^em1Cin (Cre/ERT2)^/Nju (*Lgr5*‐Cre‐ER^T2^, purchased by GemPharmatech Co., Ltd) mouse line to generate *Lgr5*‐Cre; *TSP50* ^fl/+^ mice. Homozygous *Lgr5*‐Cre; *TSP50* ^fl/fl^ mice (referred as *Lgr5* ^Cre^ *TSP50* ^fl/fl^ mice) were generated by crossing *Lgr5*‐Cre; *TSP50* ^fl/+^ mice (referred as *Lgr5* ^Cre^ *TSP50* ^fl/+^ mice). Offspring were examined by PCR analyses of tail DNA for the presence of Lgr5‐Cre‐ER^T2^ and floxed‐TSP50.

Then adult *Lgr5* ^Cre^ *TSP50* ^fl/fl^ mice and littermates mice (*Lgr5* ^Cre^ *TSP50* ^fl/+^, *TSP50* ^fl/fl^ mice) were treated with tamoxifen at a dose of 0.4 mg kg^−1^ via intraperitoneal injection for 5 days before the start of the experiment.

### Human IBD Sample Analysis

To investigate the expression of TSP50 in the intestine and Inflammatory bowel disease (IBD), paraffin‐embedded samples of UC (*n* = 12), UC samples and adjacent normal colon tissues were obtained from the Department of Pathology, Second Hospital of Jilin University. Patient information is detailed in Table S1 (Supporting Information). The research protocol was approved by the Institutional Review Board of the Second Hospital of Jilin University, and written informed consent was obtained from all patients. The diagnosis of UC was confirmed by pathologists following surgical procedures.

### Induction of DSS‐Induced IBD

Acute colitis was induced in adult male mice by administering 3% DSS (molecular weight: 35–50 kDa) in their drinking water for a duration of 7 days. Subsequently, the drinking water was switched back to regular water. Throughout the process, the mortality rate of the mice and their body weight were monitored and recorded.

### Histologic Scoring

Histologic scoring was performed on HE stained colonic tissue as follows (modified from^[^ ^1^ ^]^). The scoring system included the assessment of various parameters: inflammatory cell infiltration (score range: 0–4), goblet cell depletion or decreased mucus accumulation (score range: 0–4), mucosa thickening (score range: 0–4), and loss of crypts (score range: 0–4). The individual scores for each parameter were summed to obtain a total score (maximum score: 16).

### Plasmid Construction

The pcDNA3.1‐TSP50 was prepared previously.^[^ ^8^ ^]^ To construct pcMV‐myc‐TGFβRI and pcMV‐myc‐TGFβRII plasmids, human TGFβRI and TGFβRII cDNA were cloned into a pcMV vector containing the gene region encoding the c‐Myc epitopes.

### Cell Culture and Plasmid Transfection

HEK 293T and NCM 460 cell lines were obtained from the cell library of the Chinese Academy of Sciences. All cell lines were cultured in DMEM medium supplemented with 10% FBS, 100 U mL^−1^ penicillin, and 100 mg mL‐ streptomycin at 37 °C in a 5% CO_2_ incubator.

Upon reaching 80% confluence, a transfection procedure was conducted. Specifically, 200 µL of RPMI 1640 medium was mixed with 5 µL of X‐tremeGENE HP (Roche) and 2.5 µg of plasmid DNA. The transfection mixture was then added to each well containing the cultured cells in medium after incubation at room temperature for 30 min.

### RNA Extraction and PCR

The mice were anesthetized using isoflurane and subsequently euthanized for sampling purposes. The intestines were removed and rinsed with cold PBS. They were then cut to the same length and homogenized in Trizol (Invitrogen, Carlsbad, CA, USA). Total RNA was extracted from the homogenized samples, followed by cDNA synthesis. The cDNA was then amplified by PCR using primers specific for Muc2, Muc5B, TSP50, and β‐actin, following the methods previously described in the study.^[^ ^37^ ^]^

*Primer Sequences*:

TSP50‐F: 5′‐ACAGGGAGGAGTTCTGCTATGAGATAAC‐3′

TSP50‐R: 5′‐AAAGATGGGTGGGGCCTCGCTCTTCTTG‐3′

Muc2‐F: 5′‐CTGACCAAGAGCGAACACAA‐3′

Muc2‐R: 5′‐CATGACTGGAAGCAACTGGA‐3′

Muc5B‐F: 5′‐ CCCGTGTTGTCATCAAGGC‐3′

Muc5B‐R: 5′‐ CAGGTCTGGTTGGCGTATTTG‐3′

βactin‐F: 5′‐CGTGCGTGACATTAAGGAGAAG‐3′

βactin‐R: 5′‐GGAAGGAAGGCTGGAAGAGTG‐3′

### Western Blotting Analysis

The intestinal tissue was washed with ice‐cold PBS, then cut and weighed before being lysed in ice‐cold lysis buffer. Protein extracts were subjected to analysis by SDS‐PAGE and Western blotting. For SDS‐PAGE, 20 µg of total protein per sample was resolved, and the separated proteins were transferred onto PVDF membranes (Bio‐Rad Laboratories Inc). Immunoblotting was conducted using primary antibodies at dilutions recommended by the manufacturer, with GAPDH serving as a loading control. The immunoblots were detected using an ECL advanced Western blotting detection kit (Invitrogen). The quantification of the Western blotting results from independent repeated experiments was performed using ImageJ software.

### Immunofluorescence (IF) Analysis

The mice were anesthetized using isoflurane and subsequently euthanized for sampling purposes. The intestines were removed and intraluminally flushed once with cold PBS. They were then fixed in 4% paraformaldehyde for 12 h at 4 °C. Following fixation, the tissues were soaked in PBS containing 30% sucrose overnight at 4 °C. Subsequently, the tissues were embedded in optimal cutting temperature compound (OCT), frozen, and cut into 5 µm sections. The frozen sections were washed with PBS, and antigen retrieval was performed by immersing the sections in 0.01 m sodium citrate buffer (pH 6.0) at 98 °C for 5 min. After antigen retrieval, the sections were blocked with 2% BSA in 0.2% Triton X‐100/PBS for 1 h and then incubated with primary antibodies overnight at 4 °C. Following five washes with PBS, the sections were incubated with appropriate fluorochrome‐conjugated secondary antibodies for 1 h, followed by another five washes. Coverslips were mounted onto glass slides using 75% glycerol as a mounting medium. Images were acquired using a laser scanning confocal microscope (Zeiss, Germany).

### Hematoxylin and Eosin (HE) Staining

The mice were anesthetized using isoflurane and subsequently euthanized for sampling purposes. The intestines were removed, intraluminally flushed once with cold PBS, and then fixed in a formaldehyde solution for 48 h. Following fixation, the tissues were dehydrated using a series of graded ethanol solutions, embedded in paraffin, and sectioned into 5 µm slices. The paraffin sections were then dewaxed using xylene to remove the paraffin. Hematoxylin staining was performed for 6 min, followed by eosin staining for 2.5 min using a slide staining device. The stained sections were dehydrated, made transparent, and covered with coverslips using neutral balsam as a mounting medium. Light images of the stained sections were captured using a Nikon Eclipse microscope (Nikon, Japan).

### Immunohistochemical (IHC) Analysis

Formalin‐fixed, paraffin‐embedded (FFPE) tissue samples were cut into 5 µm sections. The paraffin was subsequently removed from the sections using xylene. To retrieve the antigens, the deparaffinized and rehydrated sections were immersed in 0.01 m sodium citrate buffer (pH 6.0) and subjected to high‐pressure treatment for 3 min. After antigen retrieval, the slides were incubated with the primary antibody for 4 h at room temperature, followed by washing with PBS and subsequent incubation with a secondary antibody. The antibodies were detected using a DAB staining kit. Hematoxylin was used as a counterstain, and the slides were mounted with neutral balsam and coverslips. Light images of the stained sections were captured using a Nikon Eclipse microscope.

### X‐Gal Staining

The mice were anesthetized using isoflurane and subsequently euthanized. Intestinal tissues were excised and intraluminally flushed once with cold PBS. The tissues were then fixed using a stationary liquid provided in the X‐gal staining kit. The subsequent steps were carried out according to the instructions provided by Beyotime Institute of Biotechnology (Haimen, China). Light images of the stained tissues were captured using a Nikon Eclipse microscope.

### Alcian Blue and Masson Staining

The FFPE tissues or organoids were sectioned into 5 µm slices, and the paraffin from the sections was removed using xylene. Subsequently, Alcian Blue and Masson staining were conducted using kits in accordance with the manufacturer's protocols.

### Fluorescence In Situ Hybridisation

The paraffin‐embedded Carnoy‐fixed sections were subjected to dewaxing and subsequently hybridized with a general bacterial 16S rRNA probe (EUB338) at a concentration of 10 ng mL^−1^. Following hybridization, the tissues were immunostained for DNA using DAPI. The resulting images were analyzed using an Olympus BX50 fluorescence microscope.

### Mucus Thickness

After a 12‐hour fasting period, mice were anesthetized with isoflurane and killed by cervical dislocation. The colon was subjected to dissection and subsequent irrigation with physiological saline. Colonic tissues were meticulously secured within a horizontal fixation apparatus, maintaining their natural extended length. To visualize the surface of the transparent mucus, a suspension of activated charcoal particles (Kebo Lab, Huddinge, Sweden) in mannitol solution was added on the apical surface, the charcoal particles were allowed to sediment on to the mucus surface. Glass micropipettes (custom glass tubing; OD, 1.2 mm; ID, 0.6 mm; Rederick Haer, Brunswick, ME) were pulled to a tip diameter of 1–2 µm. By coupling a micropipette attached to a membrane clamp, observed through a stereomicroscope (Leica MZ l2.5), the distance between the epithelial and mucosal surfaces was quantified to ascertain the overall mucous thickness. Specifically, a custom fixation apparatus was employed to document the height of the micropipette connected to the membrane clamp under the stereomicroscope. When the micropipette's leading edge was positioned on the mucosal surface, the corresponding height measurement was recorded as H1. Maintaining a constant horizontal orientation, as the descending micropipette's leading edge made contact with the epithelial surface, the corresponding height measurement was recorded as H2. The mucous thickness at the respective position was determined by subtracting H2 from H1. Following three repetitions of measurements, the average was computed. Subsequently, the position of the micropipette was altered, and mucous thickness was measured at four distinct locations for each mouse.

### Intestinal Permeability

After overnight fasting, the mice were administered 4 kD FITC‐dextran (Sigma) diluted in PBS the following day. Four hours after intragastric administration (at a dosage of 60 mg per 100 g body weight), the fluorescence intensity in the serum was measured using excitation at 485 nm and emission at 535 nm. To ensure accurate quantification, the fluorescence intensity was normalized using FITC‐glucan standards, following the manufacturer's protocol.

To determine the levels of LPS in mouse serum, ELISA was performed according to the manufacturer's protocol provided by Mlbio. The measurements were standardized to ensure accurate and reliable quantification of LPS levels.

### Co‐Immunoprecipitation (Co‐IP)

Co‐immunoprecipitation (Co‐IP) was performed using Protein A/G Magnetic Beads (HY‐K0202, MedChemExpress, Monmouth Junction, NJ, USA) following the manufacturer's protocols to investigate protein‐protein interactions. First, 25 µL of magnetic beads pretreated with 0.5% Triton X‐100 in PBS (PBST) solution were mixed with PBST containing the antibodies at a final concentration of 5 µg mL^−1^ and incubated at 4 °C for 2 h on a flip mixer. The proteins extracted from cells using IP lysis solution (87 787, Thermo Fisher Scientific, Waltham, MA, USA) were then mixed with the antibody‐magnetic‐bead complexes and incubated overnight at 4 °C on a flip mixer (88 881 002, Thermo Fisher Scientific, Waltham, MA, USA). After thorough washing, the antigen‐antibody‐magnetic‐bead complexes were boiled in 25 µL of 1x SDS‐PAGE loading buffer, and the supernatant was used for Western blotting analysis.

### GST Pull‐Down Assay

The GST‐TSP50 protein was prepared and purified as described previously.^[^ ^7^ ^]^ Total protein extraction from cells treated according to the experimental requirements was performed using RIPA lysate. Subsequently, the target proteins were incubated overnight at 4 °C with GST pull‐down protein binding buffer, along with an equilibrated 50% gel suspension and bait proteins. After centrifugation, the supernatant was removed, and the gel was resuspended using GST pull‐down binding buffer to effectively wash away unbound proteins. Finally, GST pull‐down elution buffer was added to the gel and incubated for 10 min. Following centrifugation at 1000 g for 2 min at 4 °C, the supernatant was collected for Western blotting detection.

### Crypt Isolation and Organoid Culture

The colon was isolated and longitudinally opened, followed by removal of feces. The intestine was then washed with cold DPBS for five cycles. Subsequently, the tissue was diced into ≈5 mm pieces and washed again with cold DPBS. These tissue fragments were incubated on a shaker for 10 min on ice in 20 mL of 20 mm EDTA in DPBS. After removing the EDTA medium, the tissue fragments were further incubated on a shaker for 30 min on ice in 15 mL of 20 mm EDTA in DPBS. Gravity‐induced settling of the tissue fragments allowed for the transfer of the supernatant to a new 50 mL tube. The supernatant, containing enriched crypts, was then thoroughly suspended in cold DPBS three times. All the supernatant was consolidated into the same 50 mL tube, which was subsequently passed through a 70‐µm cell strainer to eliminate residual villous material. The isolated crypts in the supernatant were separated from single cells by centrifugation at 500 g for 5 min. The isolated crypts were washed with 5 mL of DMEM/F12 medium containing 5% FBS and then centrifuged at 500 g for 5 min to collect the crypts. Following the isolation and enumeration of murine intestinal crypts, form a mixed pellet by combining them with Matrigel in a 1:1 ratio. Subsequently, dispense 50–80 µL of the Matrigel : Crypt mixture as droplets into each well of a 24‐well plate. Incubate the plate in a 37 °C incubator to facilitate the polymerization of Matrigel for a duration of 10–15 min. Add 500 µL of IntestiCult OGM Mouse Basal Medium to each well. Surviving organoids are visible on the second day. Medium were performed changes every 2–4 days. Over the course of 5–7 days, viable intestinal stem cells should form organoids. All organoids utilized in this investigation were exclusively obtained from primary cultures of murine intestinal tissues without any subsequent passages. These organoids were derived from the intestinal tissues of mice aged 6 weeks, encompassing various genotypes.

### Statistical Analysis

Statistical analyses were performed using the GraphPad Prism software. The data are presented as means ± standard error of the mean (SEM). A two‐tailed unpaired Student's *t*‐test was employed to calculate significant differences between two groups of samples or mice, with a significance threshold of *P*< 0.05. Asterisks were used to denote the level of significance, where ns indicates not significant, * indicates *P*< 0.05, ** indicates *P*< 0.01, and *** indicates *P*< 0.001. All data presented are representative of a minimum of three independent experiments.

### Ethical Approval

All animal studies were conducted with approval from the Animal Research Ethics Committee of Northeast Normal University (NENU/IACUC, AP20191225) of China and performed in accordance with established guidelines.

## Conflict of Interest

The authors declare no conflict of interest.

## Author Contributors

J.L., C.N., and H.A. contributed equally to this work. J.L. designed, performed experiments, analyzed data, and wrote the paper. C.N., H.A., X.L., L.Z., and Y.L. performed experiments. S.W., F.G., C.Y., L.S., Y.H., L.Z., G.W., Y.S., Z.B., and X.Y. provided technical support. Y.B. conceived ideas, wrote the paper, and oversaw the research programme.

## Supporting information

Supporting Information

## Data Availability

The data that support the findings of this study are available from the corresponding author upon reasonable request.
