# Supplementary material for: TSP50 Attenuates DSS‐Induced Colitis by Regulating TGF‐β Signaling Mediated Maintenance of Intestinal Mucosal Barrier Integrity
Source: Adv Sci (Weinh). 2024 Jan 8;11(11):2305893. doi: 10.1002/advs.202305893 (PMC10953580; doi:10.1002/advs.202305893)
Supplement: Supplementary file 1 — Supporting Information [file ADVS-11-2305893-s001.pdf]

ADVANCED SCIENCE
Open Access

Supporting Information

for *Adv. Sci.*, DOI 10.1002/advs.202305893

TSP50 Attenuates DSS-Induced Colitis by Regulating TGF-β Signaling Mediated Maintenance of Intestinal Mucosal Barrier Integrity

Jiawei Li, Chunxue Niu, Huihan Ai, Xiaoli Li, Linlin Zhang, Yan Lang, Shuyue Wang, Feng Gao, Xianglin Mei, Chunlei Yu, Luguo Sun, Yanxin Huang, Lihua Zheng, Guannan Wang, Ying Sun, Xiaoguang Yang, Zhenbo Song and Yongli Bao**

Table S1: Clinical data of patients with IBD.

**IBD patients**				
No.	Age	Sex	Organ	Pathological Diagnosis
1	56	Male	Colon	Ulcerative Colitis (UC)
2	59	Male	Colon	UC
3	60	Male	Colon	UC
4	40	Male	Colon	UC
5	38	Male	Colon	UC
6	55	Male	Colon	UC
7	41	Female	Colon	UC
8	45	Male	Colon	UC
9	51	Male	Colon	UC
10	53	Male	Colon	UC
11	43	Male	Colon	UC
12	54	Female	Colon	UC

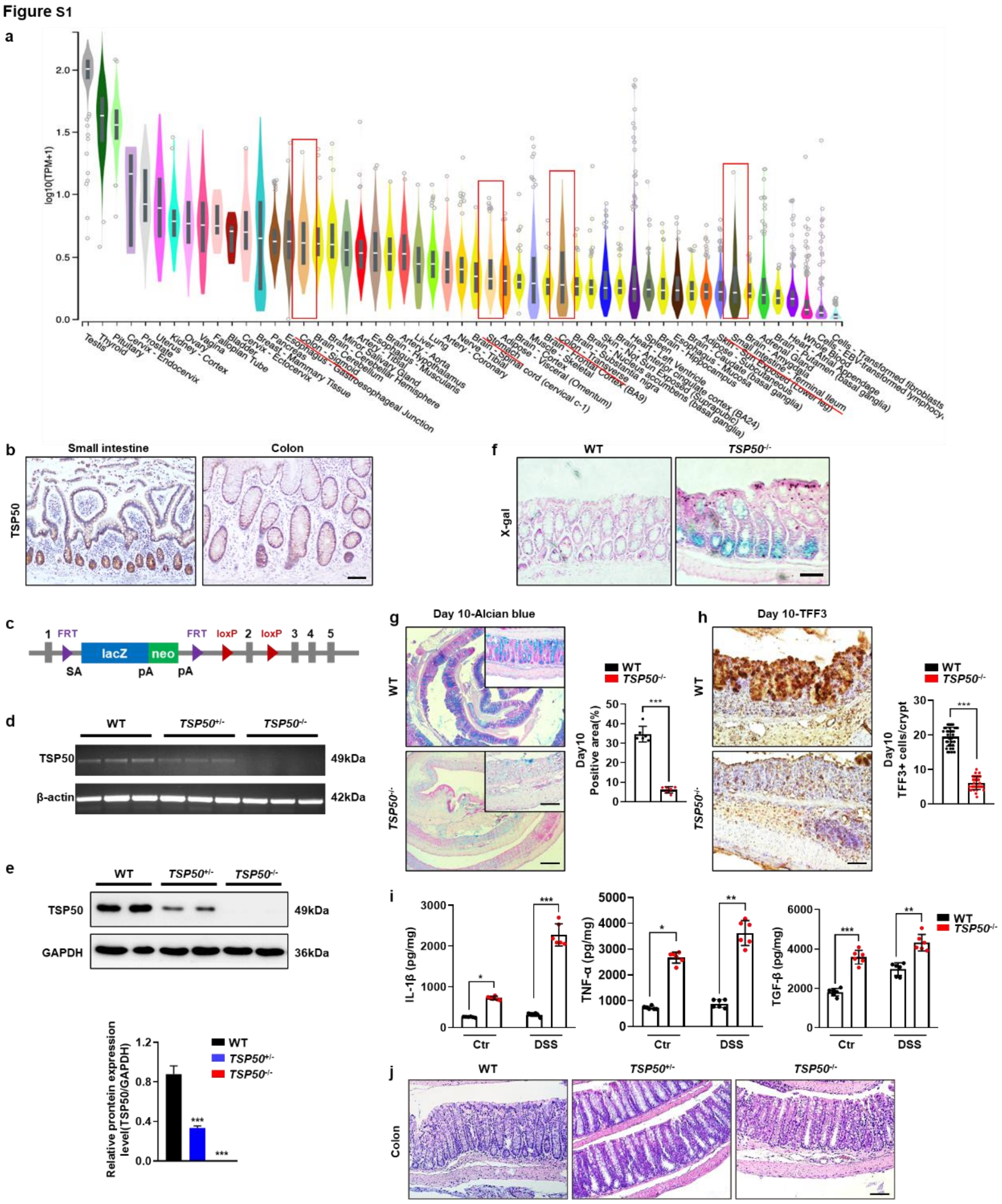

Figure S1: Expression and identification of TSP50 in the intestine.

a. The expression levels of TSP50 in human normal tissues were obtained from the GTEx database. From left to right, the tissues marked with red boxes are Colon-Sigmoid, Stomach, Colon-Transverse, and Small Intestine-Terminal Ileum.

b. Immunohistochemical analysis of TSP50 in human small intestine and colon was conducted. Scale bar: 50 μm.

c. A schematic diagram depicting the targeting strategy utilized for the generation of TSP50-deficient mice.

d. The mRNA levels of TSP50 in intestinal tissue from 6-week-old mice were assessed via PCR analysis. β-actin was employed as an internal control to verify the efficacy of cDNA synthesis and PCR amplification.

e. TSP50 levels were determined through Western blotting analysis. Colon samples were obtained from 6-week-old WT, $TSP50^{+/-}$ and $TSP50^{-/-}$ mice. GAPDH was used as loading control. (The Western blotting results were quantitatively analyzed using ImageJ software, Data are represented as the mean ± SD. n=3; ****P* < 0.001. unpaired, two-tailed Student's *t* test).

f. X-gal staining was performed on colon tissues obtained from 6-week-old mice. Scale bar: 50 μm.

g. Representative images of colon tissue slices stained with Alcian Blue were obtained from mice treated with 3% DSS on day 10 (Left). Quantification of the Alcian Blue staining results by calculating the percentage of positive staining area relative to the total tissue area (Right). (The Alcian Blue results were quantitatively analyzed using ImageJ software. Data are represented as the mean ± SD. n=6; ****P* < 0.001. unpaired, two-tailed Student's *t* test). Scale bar: Entire colon section 200 μm, top right magnification 100 μm.

h. The TFF3 antibody was employed in immunohistochemistry to assess the goblet cell count in the colon of mice on day 10 following treatment with 3% DSS (Left). Quantification of TFF3-positive cells in each crypt by counting ≥6 crypts in each mouse (Right). (The immunohistochemistry results were quantitatively analyzed using ImageJ software. Data are represented as the mean ± SD. n=6; ****P* < 0.001. unpaired, two-tailed Student's *t* test). Scale bar:

50 μm.

i. The expression levels of IL-1β, TNF-α, and TGF-β in the colon tissues of WT and *TSP50*$^{-/-}$ mice were measured using ELISA. The experimental groups included Ctr (non-induced with DSS) and DSS (induced with DSS). (Data are represented as the mean ± SD. n=3; *$P < 0.05$. **$P < 0.01$. unpaired, two-tailed Student's *t* test).

j. Representative images of colon HE staining from WT, *TSP50*$^{+/-}$and *TSP50*$^{-/-}$ mice at 6 weeks of age.

All data are representative of at least three independent experiments.

Figure S2

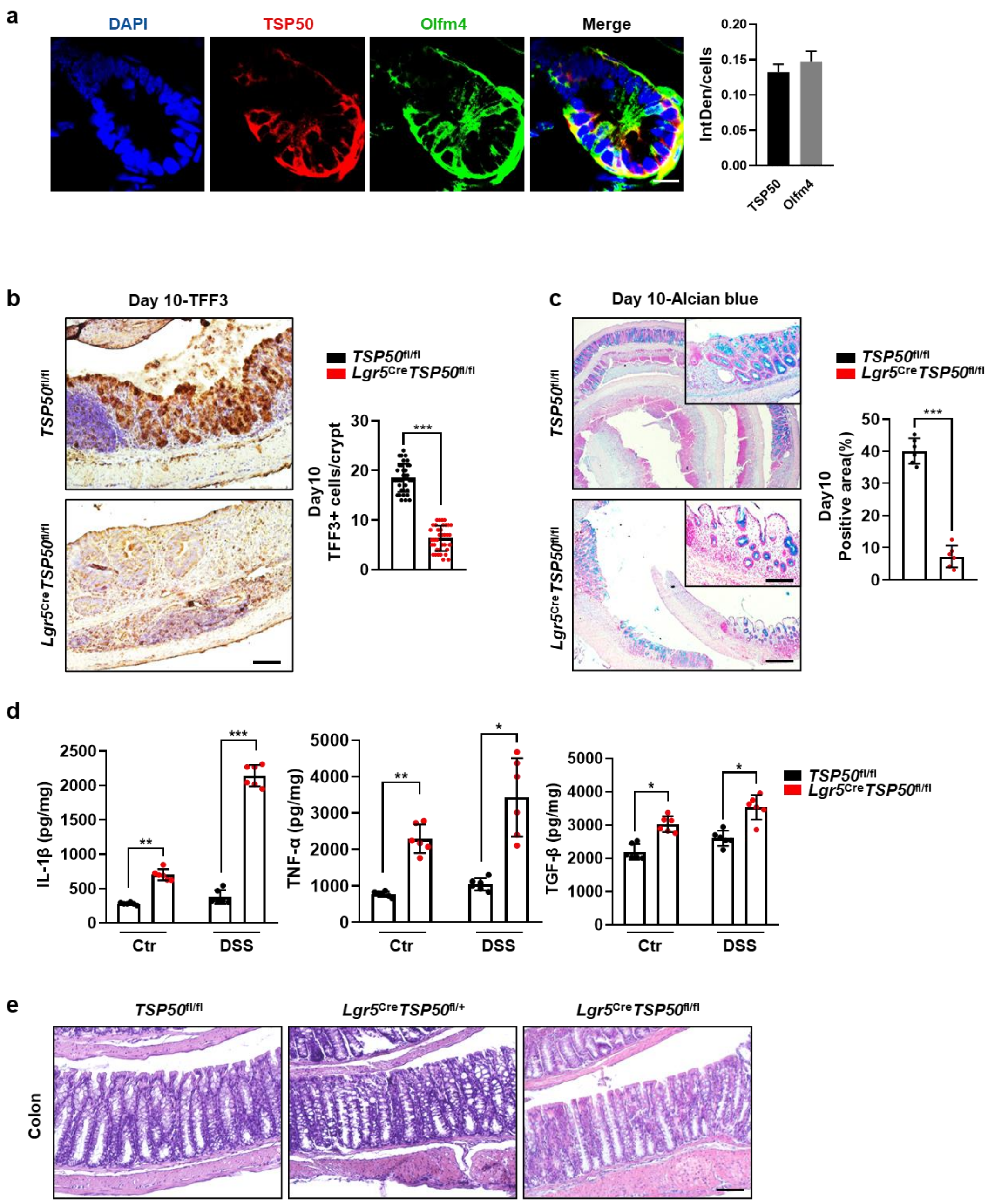

Figure S2: Increased susceptibility to colitis in mice with TSP50 deficiency in ISCs.

a. Immunofluorescence co-localization of TSP50 with Olfm4 in the intestinal tract of 6-week-old wild-type mice (Left). Quantification of the immunofluorescence staining results by calculating the integrated density (IntDen) divided by the cell count (DAPI count) (Right). (The immunofluorescence results were quantitatively analyzed using ImageJ software. Data are represented as the mean ± SD). Scale bar: 10 μm.

b. The TFF3 antibody was employed in immunohistochemistry to assess the goblet cell count in the colon of mice on day 10 following treatment with 3% DSS (Left). Quantification of TFF3-positive cells in each crypt by counting ≥6 crypts in each mouse (Right). (The immunohistochemistry results were quantitatively analyzed using ImageJ software. Data are represented as the mean ± SD. n=6; ***$P < 0.001$. unpaired, two-tailed Student's *t* test). Scale bar: 50 μm.

c. Representative images of colon tissue slices stained with Alcian Blue were obtained from mice treated with 3% DSS on day 10 (Left). Quantification of the Alcian Blue staining results by calculating the percentage of positive staining area relative to the total tissue area (Right). (The Alcian Blue results were quantitatively analyzed using ImageJ software. Data are represented as the mean ± SD. n=6; ***$P < 0.001$. unpaired, two-tailed Student's *t* test). Scale bar: Entire colon section 200 μm, top right magnification 100 μm.

d. The expression levels of IL-1β, TNF-α, and TGF-β in the colon tissues of $TSP50^{fl/fl}$ and $Lgr5^{Cre}TSP50^{fl/fl}$ mice were measured using ELISA. The experimental groups included Ctr (non-induced with DSS) and DSS (induced with DSS). (Data are represented as the mean ± SD. n=3; *$P < 0.05$. **$P < 0.01$. ***$P < 0.001$. unpaired, two-tailed Student's *t* test).

e. Representative images of colon HE staining from $TSP50^{fl/fl}$, $Lgr5^{Cre}TSP50^{fl/+}$ and $Lgr5^{Cre}TSP50^{fl/fl}$ mice at 6 weeks of age.

All data are representative of at least three independent experiments.

Figure S3

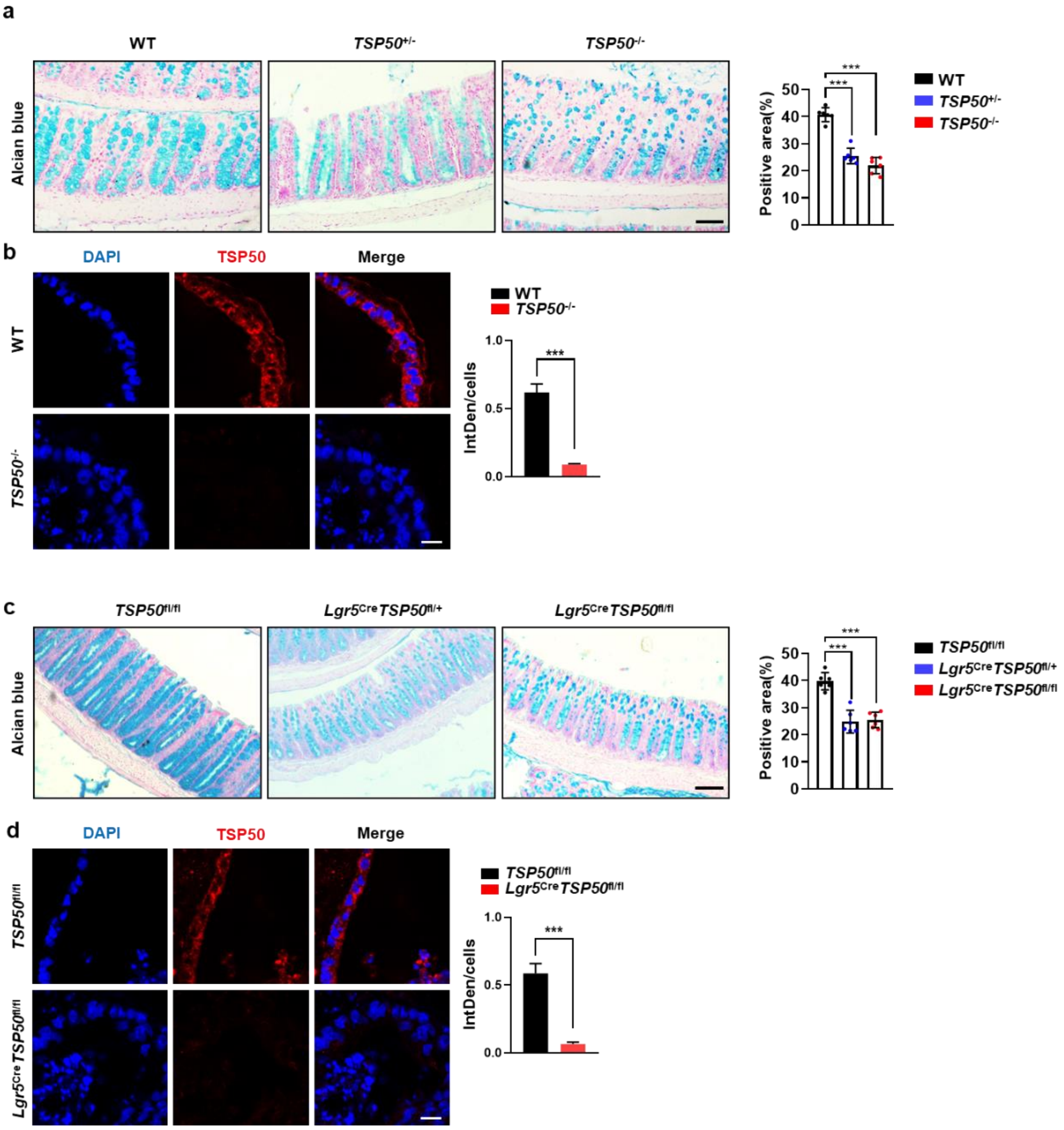

Figure S3: Detection of TSP50 expression in organoids.

a. Representative images of colon tissue slices stained with Alcian Blue were acquired from 6-week-old mice (Left). Quantification of the Alcian Blue staining results by calculating the percentage of positive staining area relative to the total tissue area (Right). (The Alcian Blue results were quantitatively analyzed using ImageJ software. Data are represented as the mean ± SD. n=6; ****P* < 0.001. unpaired, two-tailed Student's *t* test). Scale bar: 50 μm.

b. Immunofluorescence staining was utilized to assess the expression of TSP50 in the organoids cultured for 7 days from 6-week-old WT and *TSP50*$^{-/-}$ mice (Left). Quantification of the

immunofluorescence staining results by calculating the integrated density (IntDen) divided by the cell count (DAPI count) (Right). (The Immunofluorescence results were quantitatively analyzed using ImageJ software.Data are represented as the mean ± SD. n=3; ***P < 0.001. unpaired, two-tailed Student’s *t* test). Scale bar: 10 µm.

c. Representative images of colon tissue slices stained with Alcian Blue were acquired from 6-week-old mice (Left). Quantification of the Alcian Blue staining results by calculating the percentage of positive staining area relative to the total tissue area (Right). (The Alcian Blue results were quantitatively analyzed using ImageJ software. Data are represented as the mean ± SD. n=6; ***P < 0.001. unpaired, two-tailed Student’s *t* test). Scale bar: 50 µm.

d. Immunofluorescence staining was utilized to assess the expression of TSP50 in the organoids cultured for 7 days from 6-week-old $TSP50^{fl/fl}$ and $Lgr5^{Cre}TSP50^{fl/fl}$ mice (Left). Quantification of the immunofluorescence staining results by calculating the integrated density (IntDen) divided by the cell count (DAPI count) (Right). (The Immunofluorescence results were quantitatively analyzed using ImageJ software. Data are represented as the mean ± SD. n=3; ***P < 0.001. unpaired, two-tailed Student’s *t* test). Scale bar: 10 µm.

All data are representative of at least three independent experiments.

Figure S4

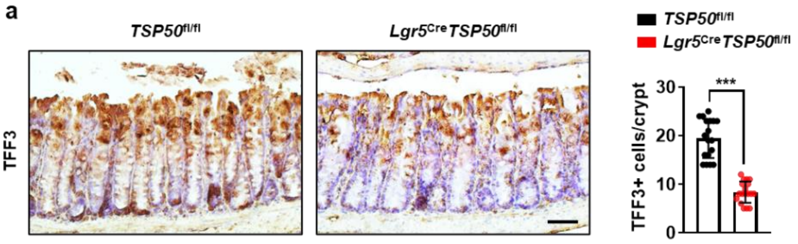

Figure S4: Detection of mucin TFF3 expression in the mouse colon.

a. The TFF3 antibody was employed in immunohistochemistry to assess the goblet cell count in the colon of 6-week-old mice (Left). Quantification of TFF3-positive cells in each crypt by counting ≥6 crypts in each mouse (Right). (The TFF3 results were quantitatively analyzed using ImageJ software, Data are represented as the mean ± SD. n=6; ****P* < 0.001. unpaired, two-tailed Student's *t* test). Scale bar: 50 µm.

All data are representative of at least three independent experiments.

Figure S5

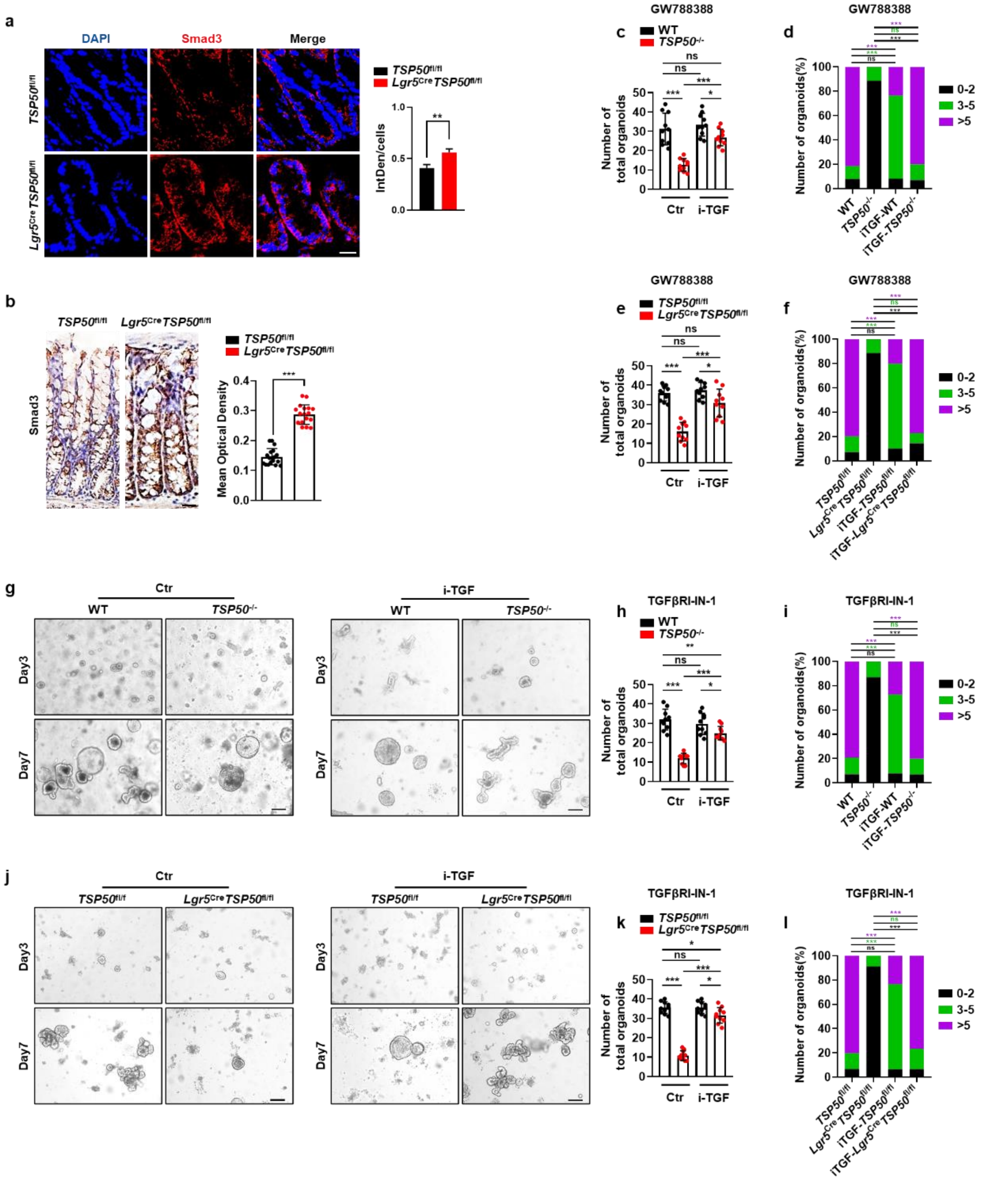

Figure S5: TSP50 deficiency results in aberrant activation of the TGF-β signaling pathway.

a. The expression of Smad3 was evaluated by immunofluorescence in the colon tissues of 6-week-old *TSP50*$^{fl/fl}$ and *Lgr5*^{Cre}*TSP50*$^{fl/fl}$ mice (Left). Quantification of the immunofluorescence staining results by calculating the integrated density (IntDen) divided by the cell count (DAPI count) (Right). (The Immunofluorescence results were quantitatively analyzed using ImageJ

software.Data are represented as the mean ± SD. n=3; ns, not significant. ***P < 0.001. unpaired, two-tailed Student's t test). Scale bar :20 μm.

b. The expression of Smad3 was evaluated by immunohistochemical staining in the colon tissues of 6-week-old *TSP50*$^{fl/fl}$ and *Lgr5*^{Cre}*TSP50*$^{fl/fl}$ mice (Left). The mean optical density in immunohistochemical staining images of Smad3 was measured by ImageJ software (Right). (The immunohistochemical results were quantitatively analyzed using ImageJ software, Data are represented as the mean ± SD. n=6, ***P < 0.001. unpaired, two-tailed Student's t test). Scale bar :20 μm.

c, e, h, k. The number of colon organoids formed in 6-week-old mice on day 7 of culture was determined. (Data are represented as the mean ± SD. n=10; ns, not significant. *P < 0.05. **P < 0.01.***P < 0.001. unpaired, two-tailed Student's t test).

d, f, i, l. The percentage of organoids with varying numbers of buds was quantified on day 7 of the organoid culture. Organoid buds number 0-2, black; Organoid buds number 3-5, green; Organoid buds number >5, purple. (Data are represented as the mean ± SD. n=10; ns, not significant. ***P < 0.001. unpaired, two-tailed Student's t test).

c, d correspond to Fig. 5g; e, f correspond to Fig. 5f; h, i correspond to Fig. S5g; k, l correspond to Fig. S5j.

g. Representative morphological images of colon organoid cultures from 6-week-old WT and *TSP50*$^{-/-}$ mice on the 3rd and7th day of culture. The cultures were treated with the control agent (Ctr) or the TGF-β signaling pathway inhibitor TGFβRI-IN-1 (i-TGF) at a concentration of 5μM. Scale bar :100 μm.

j. Representative morphological images of colon organoid cultures from 6-week-old

TSP50$^{fl/fl}$ and *Lgr5*^{Cre}*TSP50*$^{fl/fl}$ mice on the 3rd and 7th day of culture. The cultures were treated with the control agent (Ctr) or the TGF-β signaling pathway inhibitor TGFβRI-IN-1 (i-TGF) at a concentration of 5μM. Scale bar :100 μm.

All data are representative of at least three independent experiments.

Figure S6

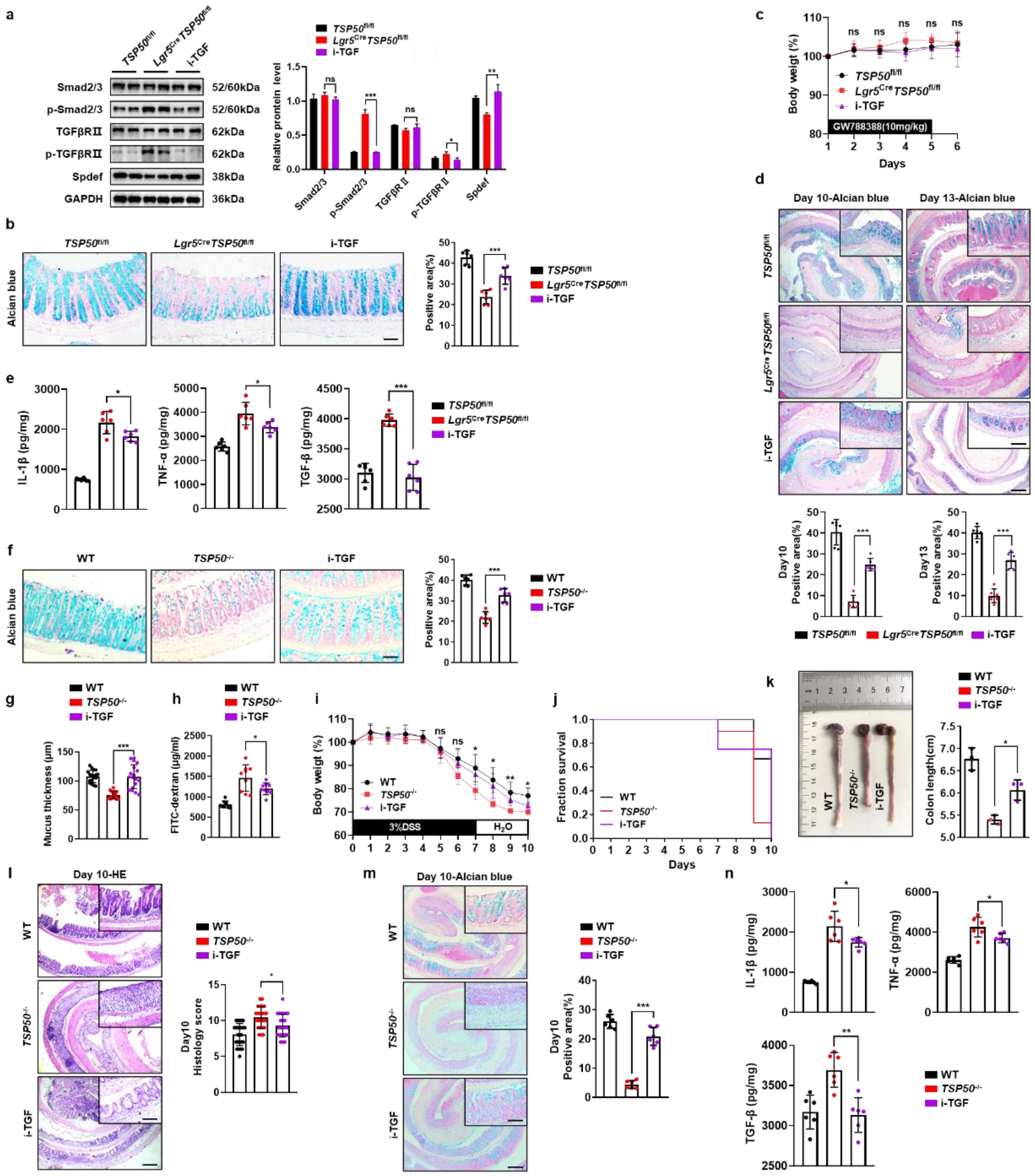

Figure S6: Restoring intestinal barrier function and alleviating colitis through TGF-β signaling inhibition in TSP50-deficient mice.

a. Following 5-day oral administration of a TGF-β inhibitor (i-TGF: 10 mg/kg) or control agent, the expression levels of TGF-β signaling-related proteins, including Smad2/3, p-Smad2/3, TGFβRII, p- TGFβRII and Spdef in the colon tissues of 6-week-old *TSP50*$^{fl/fl}$ and *Lgr5*^{Cre}*TSP50*$^{fl/fl}$ mice were assessed by Western blotting. GAPDH was used as loading control. (The Western blotting results were quantitatively analyzed using ImageJ software, Data are represented as the

mean ± SD. n=3; ns, not significant. **P* < 0.05. ***P* < 0.01.****P* < 0.001. unpaired, two-tailed Student's *t* test).

b. Representative Alcian Blue staining of goblet cells in colon tissues of *TSP50*$^{fl/fl}$ and *Lgr5*^{Cre}*TSP50*$^{fl/fl}$ mice, following 5-day oral administration of a TGF-β inhibitor (i-TGF: 10 mg/kg) or control agent (Left). Quantification of the Alcian Blue staining results by calculating the percentage of positive staining area relative to the total tissue area (Right). (The Alcian Blue results were quantitatively analyzed using ImageJ software. Data are represented as the mean ± SD. n=6; ****P* < 0.001. unpaired, two-tailed Student's *t* test). Scale bars: 50 µm.

c. After 5 days of gavage with TGF-β inhibitor (i-TGF: 10 mg/kg) or the control agent, the percentage change in body weight of the mice was assessed. (Data are represented as the mean ± SD, *TSP50*$^{fl/fl}$, n=6; *Lgr5*^{Cre}*TSP50*$^{fl/fl}$, n=6; i-TGF, n=7; ns, not significant).

d. After a 5-day oral administration of a TGF-β inhibitor (i-TGF: 10 mg/kg) or control agent, colitis was induced using 3% DSS. On the 10th and 13th days of colitis induction, representative images of colon tissue sections were obtained following staining with Alcian Blue (Top). Quantification of the Alcian Blue staining results by calculating the percentage of positive staining area relative to the total tissue area (Right). (The Alcian Blue results were quantitatively analyzed using ImageJ software. Data are represented as the mean ± SD. n=6; ****P* < 0.001. unpaired, two-tailed Student's *t* test). Scale bar: Entire colon section 200 µm, top right magnification 100 µm.

e. After 5 days of oral administration of a TGF-β inhibitor (i-TGF: 10 mg/kg) or control agent, colitis was induced using 3% DSS. The levels of IL-1β, TNF-α, and TGF-β in colonic tissue were measured. (Data are represented as the mean ± SD. n=3; **P* < 0.05. ****P* < 0.001. unpaired,

two-tailed Student's *t* test).

f. Representative Alcian Blue staining of goblet cells in colon tissues of *TSP50*$^{-/-}$ mice, following 5-day oral administration of a TGF-β inhibitor (i-TGF: 10 mg/kg) or control agent (Left). Quantification of the Alcian Blue staining results by calculating the percentage of positive staining area relative to the total tissue area (Right). (The Alcian Blue results were quantitatively analyzed using ImageJ software. Data are represented as the mean ± SD. n=6; ***P < 0.001. unpaired, two-tailed Student's *t* test). Scale bars: 50 μm.

g. Total mucus thickness in the distal colon of WT and *TSP50*$^{-/-}$ mice (with or without inhibitor) was measured after 5-day oral administration of a TGF-β (i-TGF: 10 mg/kg) inhibitor or control agent. (Data are represented as the mean ± SD. n=6; ***P < 0.001. unpaired, two-tailed Student's *t* test).

h. Intestinal permeability was assessed by measuring the concentration of FITC-dextran in the serum of WT and *TSP50*$^{-/-}$ mice after treatment with a TGF-β inhibitor or control agent. (Data are represented as the mean ± SD. n=6; *P < 0.05. unpaired, two-tailed Student's *t* test).

i, j. The percentage of body weight loss (i) and the survival rate (j) of WT and *TSP50*$^{-/-}$ mice (with or without TGF-β inhibitor) was shown after 5-day oral administration of a TGF-β inhibitor (i-TGF: 10 mg/kg) or control agent, followed by induction of colitis using 3% DSS. (Data are represented as the mean ± SD, WT, n=6; *TSP50*$^{-/-}$, n=6; i-TGF, n=6; *TSP50*$^{-/-}$ as i-TGF control. ns, not significant. *P < 0.05. **P < 0.01).

k. After a 5-day oral administration of a TGF-β inhibitor (i-TGF: 10 mg/kg) or control agent, colitis was induced using 3% DSS. On the 10th day of colitis induction, colonic length was measured. (Data are represented as the mean ± SD. n=3; *P < 0.05. unpaired, two-tailed Student's

t test).

l, m. After a 5-day oral administration of a TGF-β inhibitor (i-TGF: 10 mg/kg) or control agent, colitis was induced using 3% DSS. On the 10th days of colitis induction, representative images of colon tissue sections were obtained following staining with HE (l) and Alcian Blue (m). Histological scores were evaluated in WT and *TSP50*$^{-/-}$ mice on day 10 post-DSS treatment (l, Right). Quantification of the Alcian Blue staining results by calculating the percentage of positive staining area relative to the total tissue area (m, Right). (Data are represented as the mean ± SD. n=3; *P < 0.05. ***P < 0.001. unpaired, two-tailed Student's *t* test). Scale bar: Entire colon section 200 μm, top right magnification 100 μm.

n. After 5 days of oral administration of a TGF-β inhibitor (i-TGF: 10 mg/kg) or control agent, colitis was induced using 3% DSS. The levels of IL-1β, TNF-α, and TGF-β in colonic tissue were measured. (Data are represented as the mean ± SD. n=3; *P < 0.05. **P < 0.01. unpaired, two-tailed Student's *t* test).
